# Efficient Nickel and Cobalt Recovery by Metal–Organic Framework-Based Mixed Matrix Membranes (MMM-MOFs)

**DOI:** 10.1021/acssuschemeng.4c03427

**Published:** 2024-07-31

**Authors:** Amira Nour, Waseem Iqbal, Javier Navarro-Alapont, Jesús Ferrando-Soria, Pietro Magarò, Rosangela Elliani, Antonio Tagarelli, Carmine Maletta, Teresa F. Mastropietro, Emilio Pardo, Donatella Armentano

**Affiliations:** † Dipartimento di Chimica e Tecnologie Chimiche (CTC), Università della Calabria, Rende 87036, Italy; ‡ Instituto de Ciencia Molecular (ICMol), Universidad de Valencia, Valencia 46980, Spain; § Dipartimento di Ingegneria Meccanica, Energetica e Gestionale, Università della Calabria, Rende, Cosenza 87036, Italy

**Keywords:** lithium-ion batteries, metal recovery, mixed-matrix membranes, cobalt, nickel

## Abstract

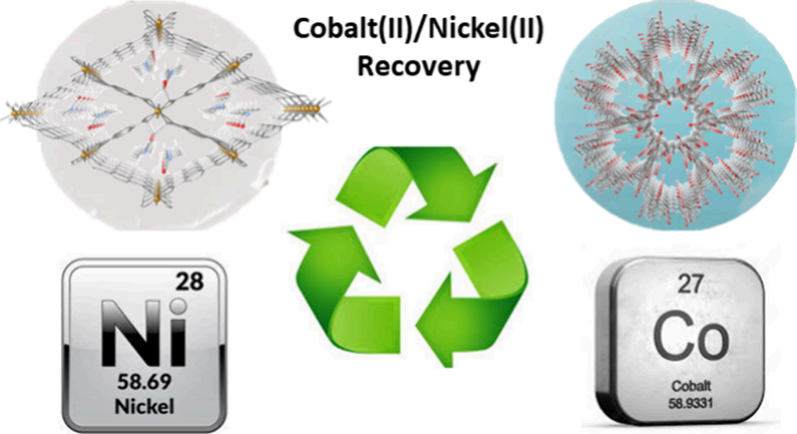

Green energy transition has supposed to give a huge boost to the electric vehicle rechargeable battery market. This has generated a compelling demand for raw materials, such as cobalt and nickel, which are key common constituents in lithium-ion batteries (LIBs). However, their existing mining protocols and the concentrated localization of such ores have made cobalt and nickel mineral conundrums, and their supplies experience shortages, which threaten to slow the progress of the renewable energy transition. Aiming to contribute to the sustainable recycling of these valuable metals from LIBs and wastewater, in this work, we explore the use of four mixed matrix membranes (MMMs) embedding different metal–organic frameworks (MOFs), i.e., **MIL-53(Al)**, **MIL-53(Fe)**, **MIL-101(Fe)**, and {Sr^II^Cu^II^_6_[(*S*,*S*)-serimox]_3_(OH)_2_(H_2_O)}·39H_2_O (**SrCu**_**6**_**Ser**) in polyether sulfone (PES), for the recovery of cobalt(II) and nickel(II) metal cations from mixed cobalt–nickel aqueous solutions containing common interfering ions. Whereas the neat PES membrane slightly contributes to the adsorption of metal ions, showing reduced removal efficiency values of 10.2 and 9.5% for Ni(II) and Co(II), respectively, the inclusion of MOFs in the polymeric matrix substantially improves the adsorption performances. The four MOF@PES MMMs efficiently remove these metals from water, with **MIL-53(Al)@PES** being the one that presents better performance, with a removal efficiency up to 95% of Ni(II) and Co(II). Remarkably, **SrCu**_**6**_**Ser@PES** exhibits outstanding selectivity toward cobalt(II) cations compared to of nickel(II) ones, with removal efficiencies of 63.7 and 15.1% for Co(II) and Ni(II), respectively. Overall, the remarkable efficiencies, versatility, high environmental robustness, and cost-effective synthesis shown by this family of MOF@PES MMMs situate them among the best adsorbents for the extraction of this kind of contaminants.

## Abstract 2

We report a family of metal−organic framework-based mixed matrix membranes that are highly efficient in the recovery of cobalt(II) and nickel(II) metal cations.

## Introduction

The popularity of cobalt and nickel metals has experienced huge growth in recent years. This is due, to a great extent, to their key role in current rechargeable batteries, in charge of propelling the green energy transition, thus becoming strategic metals in the most important economies.^1^ However, their increase in the global demand is difficult to be backed up, considering the existing mining limitations of these metals and localized nature of their ores in unstable countries, as well as current limited resources of these metals, which may not be sufficient to supply the world market. Indeed, the modest average cobalt and nickel contents of the earth crust are estimated at 25 ppm (0.0025%) and 84 ppm (0.0084%), respectively.^2^ Thus, with such low content, it seems obvious that possessing efficient methods for their recovery/recycling is mandatory to satisfy battery market demands and, consequently, to not slow down the renewable energy transition.

Current technologies for Co and Ni recovery from Li-ion batteries include different separation methods like adsorption,^3−5^ extraction,^6^ precipitation,^7^ ion exchange processes,^8^ electrochemical methods,^9^ and biological methods.^10^ Among them, adsorption methods have shown great potential for the selective capture of these metals.^11,12^ However, despite promising adsorption advances, the selective and reversible sequestration of cobalt and nickel from complex matrices containing other metals still constitutes a daunting challenge. So, for a real-world physical implementation, still very fundamental aspects, such as finding suitable low-cost and environmentally friendly materials with high metal uptake and efficient regeneration processes, need to be addressed. Taking this taken into account, there is still much work to be done to find the best performing materials for this purpose. In addition, the selective separation of cobalt from nickel is also of major importance for the recovery of cobalt from primary or secondary sources. However, this constitutes an even greater challenge. All of this gives a general overview of the complexity of this topic.

Although polymer-based materials have shown suitable properties for application as adsorbents for metal ions, including tailorable surface functionalities, easy regeneration, and environmental compatibility,^13,14^ they generally exhibit low adsorption capacity and selectivity. The incorporation of porous nanomaterials in polymer matrices is an advantageous strategy for improving their performance.^15^ Indeed, polymers are ideal supports for the fabrication of composite materials and adsorbent membranes, which synergically combine the properties of the polymeric support and the nanoporous filler. In this context, metal–organic frameworks (MOFs) are porous crystalline materials^16−18^ that, among other properties, have already shown excellent performances in both the selective capture of certain inorganic contaminants^19^ and also the recovery of precious metals.^20^ MOFs are considered a promising and effective technology for capturing heavy metals from water due to several key advantages, such as high water and structural stability^21,22^ and microporosity, which can be easily functionalized, resulting in a wide range of pore sizes and shapes^23,24^ with the capacity of flexibility and adaptability, which could be fundamental in capturing and accommodating the targeted guest compound. However, the use of MOFs as nanocrystalline powders generates some issues, including handling problems, low suspension stability, and potential dispersion of nanoparticles in water, which make their structuration mandatory for real-world applications. In connection with this, a powerful solution has been recently materialized with the inclusion of MOFs as fillers into polymeric matrices, rendering mixed matrix membranes (MMMs) embedding MOFs (MOFs-MMMs) with high efficiency/performance for wastewater treatment.^25^

In this work, we have synthesized four polyether sulfone (PES) based MOF@PES MMMs using four previously reported well-known MOFs, i.e., **MIL-53(Al)**,^26^**MIL-53(Fe)**,^27^**MIL-101(Fe)**,^28^ and Sr^II^Cu^II^_6_[(*S*,*S*)-serimox]_3_(OH)_2_(H_2_O)}·39H_2_O (**SrCu**_**6**_**Ser**),^29^ and studied their performance in cobalt(II) and nickel(II) sequestration from mixed cobalt–nickel aqueous solutions containing common interfering ions.^30^ The decision to use these MOFs is not accidental. On one side, the MIL family possess permanent porosity, wide surface areas, ease functionalization, controlled surface charges, and high water stability.^31,32^ On top of these remarkable features, MIL MOFs have shown efficient removal properties of heavy metals from water, where their removal performance is governed/controlled by several mechanisms because both metal ions and organic linkers can serve as adsorption sites.^32^ On the other side, **SrCu**_**6**_**Ser** possess medium-sized functional channels densely decorated with Lewis-basic sites (−CH_2_OH groups),^29,33^ which are known to facilitate metal coordination.^34^ In fact, **SrCu**_**6**_**Ser** has already been used successfully for the separation of certain lanthanides in the past. Thus, these four selected MOFs represent excellent playgrounds to study the performance of the resulting MOF@PES MMMs for the capture of cobalt(II) and nickel(II) in wastewater. In this work, we investigated the performance of neat PES and obtained MOF@PES MMMs for the removal of these metal ions by exposing the adsorbent membranes to aqueous solutions containing cobalt(II) and nickel(II) metal cations and measuring the decrease in metal concentration over time. A comprehensive characterization of the membranes in term of their chemical-physical and mechanical properties has also been performed. The four MOF@PES MMMs demonstrate superior performances with respect to the neat PES membranes, and the **SrCu**_**6**_**Ser**-based membrane also exhibited remarkable selectivity toward cobalt(II) cations. Overall, the remarkable efficiencies, versatility, high environmental robustness, and cost-effective synthesis shown by this family of MOF@PES MMMs situate them among the best adsorbents for the extraction of this kind of contaminants.^35−38^

## Results and Discussion

### MMM Preparation and Physical Characterization

PES, one of the most commonly used polymers for the fabrication of microfiltration membranes, was chosen and used as a convenient polymer for the preparation of targeted MOF@PES MMMs. PES is an amorphous thermoplastic polymer containing sulfone groups in the polymer repeating unit, which mainly determine its hydrophilic properties.^39^ It has high mechanical strength and high thermal and oxidative stability. It is particularly resistant to acids and alkalis in a wide range of pH (from 2 to 12) and present a good chemical resistance to aliphatic hydrocarbons and alcohols.^40,41^ The polymer also exhibits biocompatibility and sterilization resistance. The ether linkages contribute to easy processing. Moreover, it is cheaper than SWCNTs and Matrimid 5218, which we have previously used as support materials.^42,43^

Porous membranes have been prepared by non-solvent-induced phase separation (NIPS) via phase inversion. To this end, commonly, PES is dissolved in aprotic polar solvents, such as dimethylformamide (DMF) and *N*-methyl-2-pyrrolidone, (NMP) as well as tetrahydrofuran (THF), often used as cosolvent.^44^ However, these solvents have been recognized as substances of very high concern (SVHC) and are mentioned in different lists guides, such as the Chemical Manufacturing Methods for the 21st Century Pharmaceutical Industries (CHEM 21), the Globally Harmonized System of Classification and Labeling of Chemicals (GHS), and the Registration, Evaluation, Authorisation, and Restriction of Chemicals (REACH) regulation.^45,46^

Alternatively, in this work, with the aim to improve the greener nature of the MMM-MOF fabrication protocol, we have replaced these traditional hazardous and highly toxic solvents by dimethyl sulfoxide (DMSO). DMSO is generally considered a greener and safer alternative to reduce the environmental impact of membrane preparation. It can be easily manufactured and recycled after several uses and meets the requirements for a more sustainable process.^47−49^ In addition, DMSO allows to dissolve many types of fluoropolymers and polysulfones, thanks to its similar solubility characteristics and solvent–polymer interaction as DMF, DMA, and NMP.^50,51^ Moreover, it is fully miscible with water, which represents the most used nonsolvent during membrane preparation via NIPS.

We first prepared different MOF@PES MMMs by increasing the MOF/PES ratio until reaching the maximum amount of MOF able to be loaded into the PES matrix without compromising the mechanical integrity of the MMMs. A final MOF amount corresponding to 50 wt % of the projected total MMM solid content was selected and used for the preparation of the membranes. This is a relatively high MOF loading if compared with other MMMs reported in literature, which are typically dominated by polymer performance and lower MOF loading.^52^ Nevertheless, some seminal precedents are reported in the literature,^53^ where distinct polymers—i.e., poly(vinylidene fluoride) (PVDF),^54^ poly(ethylene oxide) (PEO),^55^ styrene–butadiene (SBS) copolymer,^56^ and poly(ethylene-covinyl acetate) (EVA) copolymer^57^—have been used to prepare high-loaded MOF@MMMs. These MMMs have been used for dye separations or toxic industrial chemical uptake,^58^ generally using expeditive filtration within a syringe in simple dead-stop filtration conditions.

Although a high loading of MOF is desirable to maximize the composite functionality for the selected targeted application, this can also have some drawbacks, with the decrease of mechanical resistance of the final membrane and the increase of defects, due to the mismatch between the polymer matrix and the filler particles or particle aggregates, being the most common ones.^59^ Nevertheless, remarkably, all the MOF@PES MMMs presented in this work maintain good mechanical properties, and macroscopic defects were not visible. All of the membranes are extremely pliable and are able to deform when stretched.

**Scheme 1:**
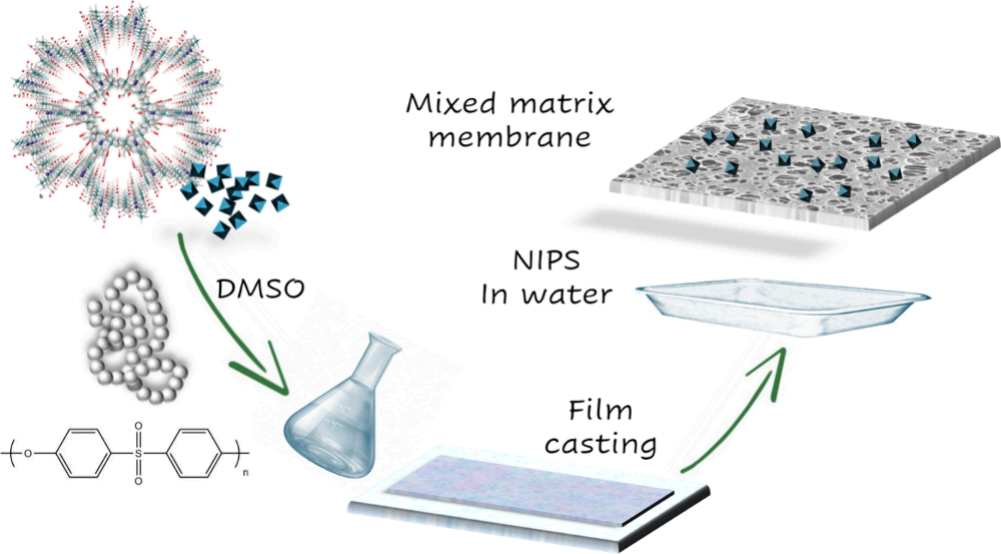
Schematic Representation of the Synthetic Approach Used for the Preparation of MMM-MOFs

**Figure 1:**
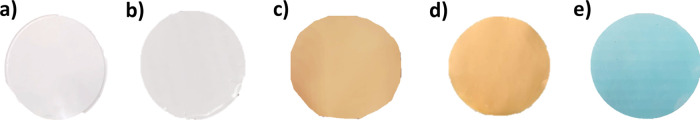
Photographs of PES (a), **MIL-53(Al)@PES** (b), **MIL-53(Fe)@PES** (c), **MIL-101(Fe)@PES** (d), and **SrCu**_**6**_**Ser@PES** (e) membranes.

MOF@PES MMMs and PES-only membrane have been prepared under the same conditions for comparison (see Scheme 1 and the Supporting Information for more details). Dealing with MOF@PES MMMs, MOFs were first dispersed in DMSO, and the suspension was gently stirred for 24 h until no aggregates of particles were visible, and the MOF particles appeared uniformly dispersed into the solvent. The dispersion was then mixed with a solution of PES in DMSO (25 wt %), which was further stirred for 24 h. Then, it was casted onto a glass substrate and immediately immersed in water, used as nonsolvent, to obtain a free-standing film having a nominal thickness of 250 μm. The membranes were washed several times to remove any trace of DMSO and then stored in ethanol. The PES membrane was prepared following the same procedure but without the MOF addition step (Figure 1).

The resulting circular membranes are characterized by an active average diameter of 47 ± 0.1 mm and an average thickness of 230 ± 2 μm. The average masses of the disks are in the ranges of 47.6 ± 2 and 70.21 ± 1.5 mg, with a resulting density of 0.12 ± 1 and 0.17 ± 1 g cm^–3^, for PES membrane and MMMs-MOFs, respectively.

**Figure 2:**
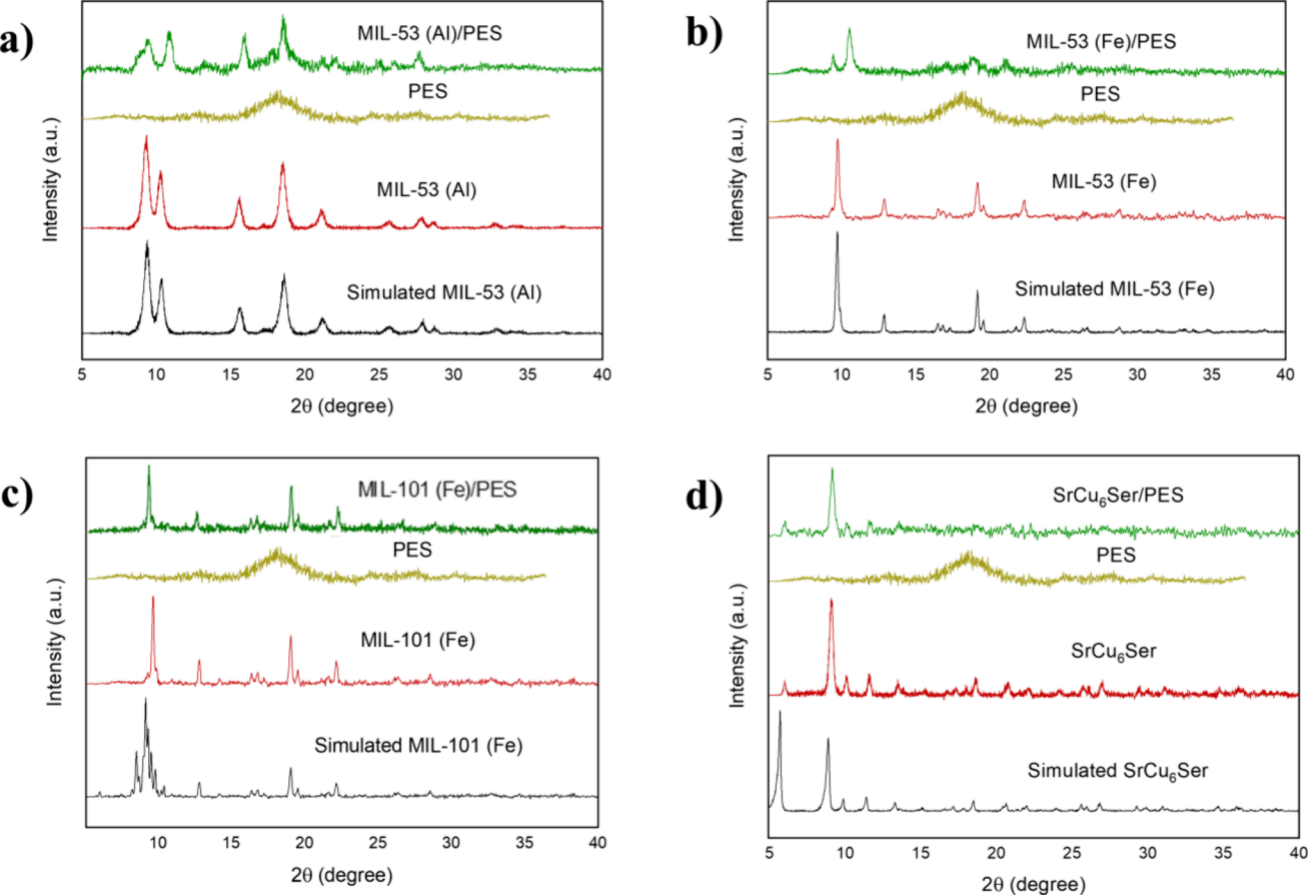
Calculated (bold lines) and experimental (solid lines) PXRD pattern profiles of synthesized MOFs after activation (red), pristine PES membrane (ochre), and MOF@PES MMMs(green). (a) **MIL-53(Al)**, (b) **MIL-53(Fe)**, (c) **MIL-101(Fe)**, and d() **SrCu**_**6**_**Ser**.

To determine whether MOF crystallinity was preserved during MMM preparation, powder X-ray diffraction experiments were performed (Figure 2). For comparison, pristine MOFs, after activation, and PES membrane were also measured. All experimental PXRD patterns of pristine activated MOFs match with the calculated ones, and a distinctive single broad curve around 18° is present for the PES membrane, indicating an amorphous phase.^60^

For the MOFs@PES MMMs, the intensity and sharpness of the peaks suggest a high degree of crystallinity of the resulting composite.^61^ Only a slight decrease of peaks' intensity compared to the MOFs is observed, which can be due to the dilution of the MOF within the amorphous polymeric matrix, in line with previously reported data.^61−63^ In particular, after loading the MOF into the MMMs, the peaks maintained the same position and profile for **MIL-53(Al)@PES**, **MIL-101(Fe)@PES** and **SrCu**_**6**_**Ser@PES**, confirming that the MOF retained its structure when added to the PES matrix.^29,62,63^ For **MIL-53(Fe)@PES** the peaks were consistent with the simulated ones, but with a shift in peaks' position at higher angles.^61^ This could be due to the characteristic “breathing” behavior of some MIL MOFs. This well-known structural flexibility can result in changes in the interplanar spacing of the MOF, leading to shifts in the peak position.

**Figure 3:**
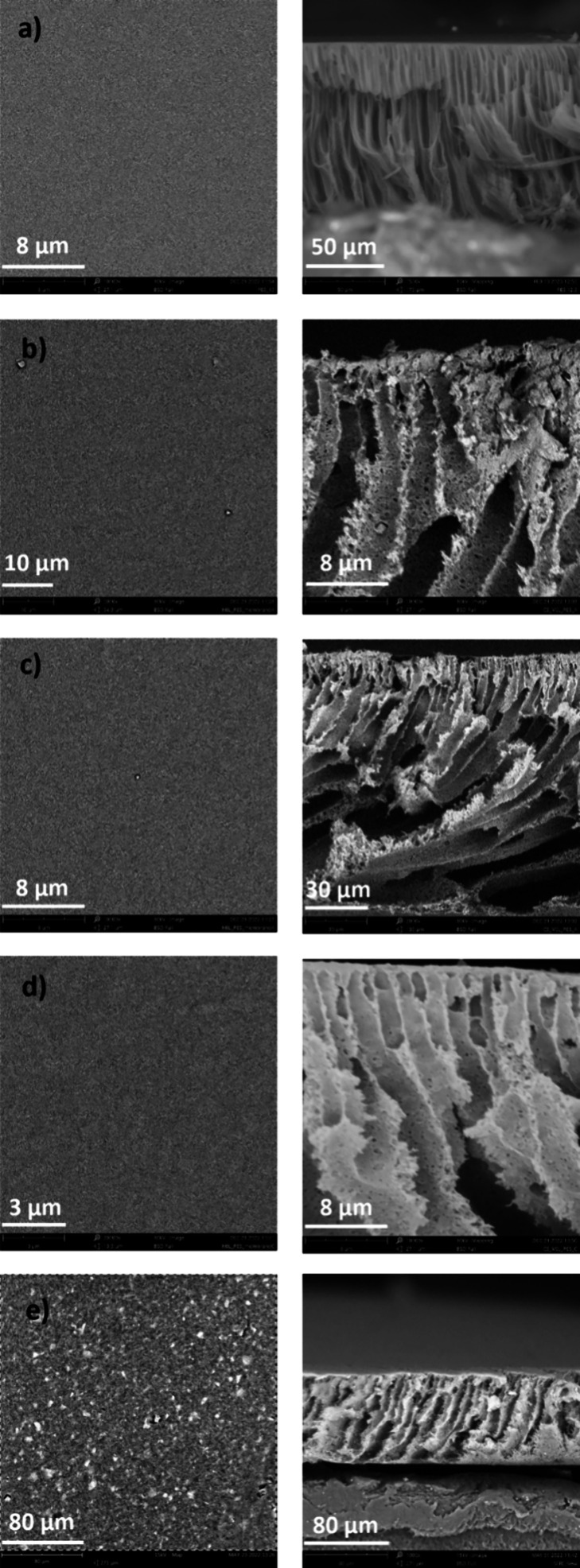
Scanning electron microscopy (SEM) images of the top surface (left) and cross section (right) of the 12.5% PES membrane (a), **MIL-53(Al)@PES** (b), **MIL-53(Fe)@PES** (c), **MIL-101(Fe)@PES** (d), and **SrCu**_**6**_**Ser@PES** (e).

Scanning-electron microscopy (SEM) images of the top and cross-section surface for the neat PES membrane (Figure 3a) evidenced an asymmetric structure composed of a thin skin layer on the top surface, a thick interlayer with smaller fingerlike voids, and a bottom layer exhibiting large finger-like voids. This morphology results from the immersion–precipitation process, where the dense skin layer is determined by the immediate polymer coagulation at the interface and the smaller and large voids by the fast DMSO and water exchange and the delay of liquid–liquid demixing during phase separation.^64^

The morphology of MOF@PES MMMs was also analyzed by SEM (Figure 3b–e and Figure S1), revealing that MOF particles are homogeneously dispersed in the polymer matrix, and their presence also modifies the texture of the resulting membrane. So, it is possible to notice the evolution of the membrane morphology from the pure finger-like structure of the neat PES membrane to a finger-like structure intermingled with a spongy-like one in MOF@PES MMMs. Interestingly, the existence of this hybrid structure stabilizes the entrapment of the MOF particles within the polymeric matrix, and no leakage of the MOF fillers was observed during the adsorption tests. In addition, MOFs’ hydrophilic nature (see below) led to the formation of larger finger-like pores because of the increased mass transfer between the solvent and the nonsolvent during phase inversion.^65^ Also, some degrees of filler agglomeration can be noticed both on the surface and in the membrane cross section, most likely determined by the phase-inversion method. As phase separation occurs during the membrane coagulation process, the MOF and polymer components tend to separate into distinct regions within the membrane according to their different hydrophilicity and mutual affinity.

Hydrophilicity of the prepared membranes was evaluated by static contact angle measurements (Figure S2) because this property highly contributes to determine the *trans-*membrane flux and its antifouling ability. Moreover, a more pronounced hydrophilicity guarantees a higher wettability and, consequently, a larger surface area for the contact and successive adsorption of metal ions present in the tested solutions. The top surface of neat PES membrane is moderately hydrophilic, with a contact angle (CA) of 69.19 ± 0.95°. Generally, MMMs-MOFs feature an increased hydrophilic character with respect to the neat PES membrane. Indeed, as expected, the addition of **MIL-53(Al)**, **MIL-53(Fe)**, **MIL-101(Fe)**, and **SrCu**_**6**_**Ser** in the MMMs effectively enhanced the hydrophilicity of the PES membrane, and the CAs decrease to 48 ± 5, 42 ± 5, 39 ± 10, and 36 ± 1°, respectively. This increase of hydrophilicity can be rationalized on the basis of two factors: (i) MOFs are intrinsically hydrophilic, determining a natural decrease of the CA of the modified membrane, and (ii) capillary effects due to the presence of hydrophilic pores.^66^

Roughness parameters (Figure S3) were obtained starting from profile scans of the top surface of the prepared membranes to reconstruct the real profile of the layer (Figure S4). The top surface of neat PES and the **MIL-53(Al)@PES** membranes exhibits the highest values of all measured roughness parameters. In particular, for the PES membrane, the measured values are *R*_a_ = 1.95 μm, *R*_q_ = 2.22 μm, and *R*_t_ = 8.35 μm, and for **MIL-53(Al)@PES**, the values are *R*_a_ = 2.35 μm, *R*_q_ = 2.71 μm, and *R*_t_ = 12.39. The addition of **MIL-53(Fe)**, **MIL-101(Fe)**, and **SrCu**_**6**_**Ser** in the MMMs seems to reduce the roughness of the membranes because for **MIL-53(Fe)@PES**, the measured values are *R*_a_ = 0.49 μm, *R*_q_ = 0.63 μm, and *R*_t_ = 4.04; for **MIL-101(Fe)@PES**, the values are *R*_a_ = 0.39 μm, *R*_q_ = 0.52 μm, and *R*_t_ = 5.21; and finally, for **SrCu**_**6**_**Ser@PES**, the values are *R*_a_ = 0.42 μm, *R*_q_ = 0.54 μm, and *R*_t_ = 4.14.

The thermal stability of both PES and MOF@PES MMMs membranes was assessed by TGA analysis. The TGA curves demonstrate that the presence of MOF of the MIL type in MOF@PES MMMs influences the thermal stability of the neat polymer decomposition, showing a shift of 10% of weight loss at 300 instead of 400 °C (Figure S5). The most appreciable variation is displayed by the **SrCu**_**6**_**Ser@PES** membrane most likely related to the bio nature of the MOF; however, it shows a very good thermal stability up to 200 °C. Accordingly, the MOF water content is reflected in a weight loss in the temperature range of 50–150 °C for MOF@PES MMMs. Above 450 °C, there is *ca*. 74% mass loss for all MOF@PES MMMs and the neat PES membrane, except for **MIL-53(Al)@PES**, which showed an 85% of weight loss. After this temperature, as expected, partial decomposition of the polymer occurred. In comparison with reported TGAs for pristine MOFs, MMMs exhibited comparable stability.^26−29^

Finally, the N_2_ adsorption isotherms at 77 K of **MIL-53(Al)**, **MIL-53(Fe)**, **MIL-101(Fe)**, and **SrCu**_**6**_**Ser** (Figure S6) confirmed a permanent porosity, which is important for carrying out successful experiments with membranes with MOFs as filler. In this sense, the calculated Brunauer–Emmett–Teller (BET) surface areas for **MIL-53(Al)**, **MIL-53(Fe)**, **MIL-101(Fe)**, and **SrCu**_**6**_**Ser** are 2894, 1209, 785, and 790 m^2^/g, respectively. These values are in agreement with reported values for all used MOFs, except for **MIL-53(Fe)** that showed a considerably improved adsorption, which most likely could be attributed to the new synthetic protocol followed in this work. The PES membrane does not show any adsorption of N_2_ at 77 K. MOF@PES MMMs present very close adsorption values to the expected ones with a 50% MOF content in the resulting MOF@PES MMMs, taking into consideration that the adsorption capacity comes from the MOF filler in the MMMs. Indeed, an average of 90% of the expected adsorption amount is reached by MOF@PES MMMs. The BET surface areas for **MIL-53(Al)@PES**, **MIL-53(Fe)@PES**, **MIL-101(Fe)@PES**, and **SrCu**_**6**_**Ser@PES** are 1302, 532, 361, and 363 m^2^ g^–1^, respectively (Figure S7). The pore size distributions of **MIL-53(Al)@PES**, **MIL-53(Fe)@PES**, **MIL-101(Fe)@PES**, and **SrCu**_**6**_**Ser@PES** are shown in Figure S8, and they are similar to those observed for the polycrystalline bulk samples,^26−29^ with the exception of **MIL-53(Fe)** that is slightly higher, in agreement with observed BET surface areas. This behavior further confirms that the crystalline structures are maintained during the formation of MMM-MOFs.

**Figure 4:**
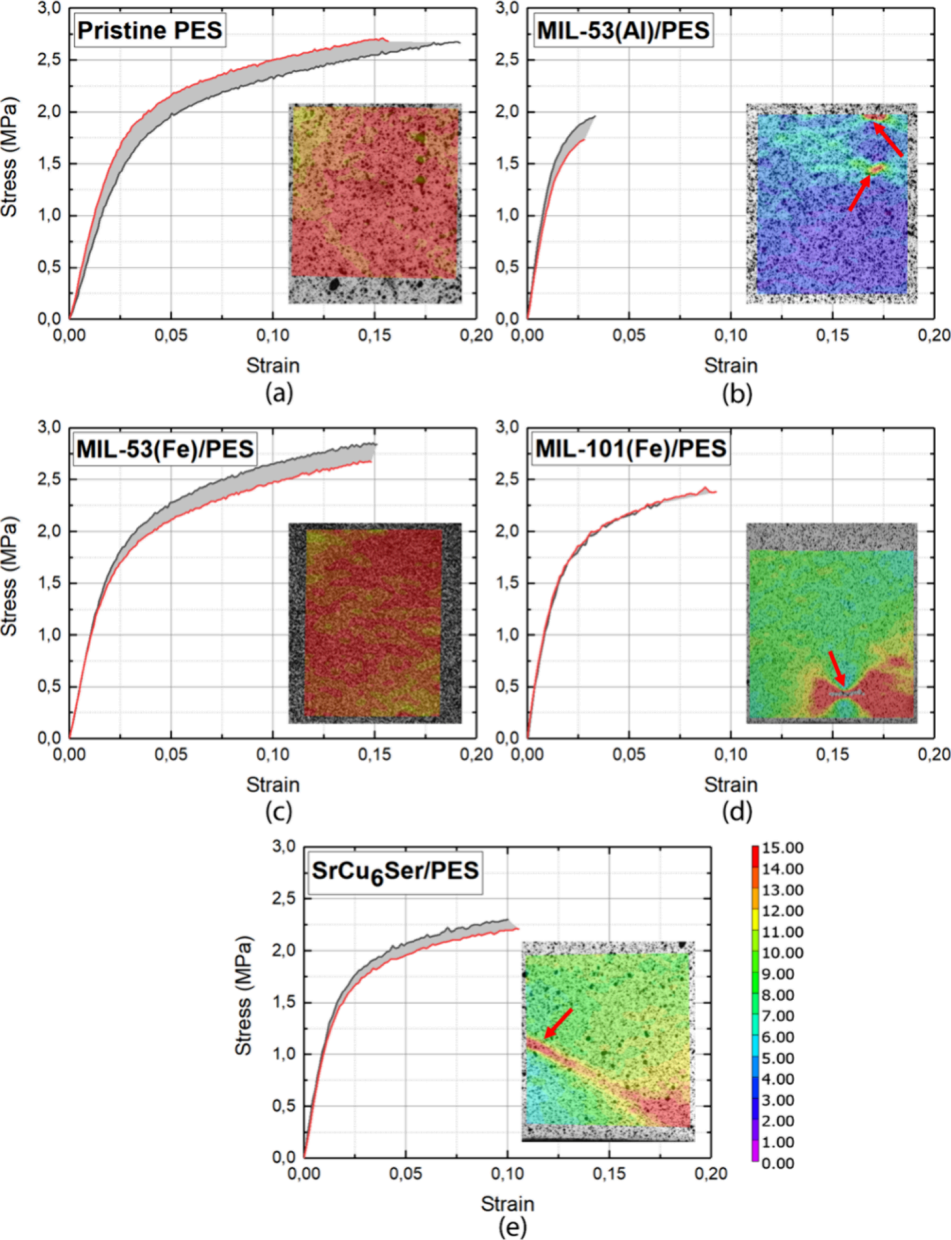
Measured engineering stress–strain (σ–ε) curves of investigated materials: (a) PES, (b) **MIL-53(Al)@PES**, (c) **MIL-53(Fe)@PES**, (d) **MIL-101(Fe)@PES**, and (e) **SrCu**_**6**_**Ser@PES**. Colored maps represent the local strain distribution before failure as obtained from digital image correlation. Arrows highlight the failure initiation locations occurring in the strain concentration zone.

The mechanical stability of membranes is a key parameter when thinking of applications. Indeed, its characterization gives important results for designing membrane filtration systems against mechanical failure. In this sense, on the one hand, we have performed macromechanical tests on the PES membrane and MOF@PES MMMs. So, displacement controlled tensile tests provided us with the measured engineering stress–strain (σ–ε) curves (Figure 4). These tests were performed in triplicate, as illustrated by the scattered bands. From them, it could be concluded that PES membrane exhibits better mechanical strength than **MIL-53(Al)@PES** in terms of both ultimate tensile strength (σ_ut_) and elongation to fracture (ε_f_). Conversely, the **MIL-53(Fe)@PES** and **SrCu**_**6**_**Ser@PES** exhibit comparable mechanical parameters to the PES membrane (Figure S9). These results are against the general assumption that the inclusion of MOF fillers has a negative effect on the strength properties of MMMs.^67^ Instead, this evidence that the mechanical properties of the filler material and its interaction mechanisms with the matrix play an important role on the whole mechanical response of the bimaterial system. *In situ* full-field strain measurements were carried out by digital image correlation for a deeper analysis of the tensile test results (insets in Figure 4). It was observed that the maximum local strain before failure is almost the same for all investigated materials, with values around 15%, which are similar to the global elongation to fracture of the PES membrane and **MIL-53(Fe)@PES**. In fact, these latter materials exhibit a mainly uniform strain distribution before failure, as shown in the DIC maps, with failure damage always initiating from the external sample edges. Conversely, differences between global and local strain were observed in the other materials with a very large mismatch in **MIL-53(Fe)@PES**. This is also associated with different failure modes with respect to PES and **MIL-53(Al)@PES**, with crack originating internally, from the near strain concentration zone (see red arrows in Figure 4). Strain concentration is attributed to local inhomogeneities due to the bimaterial nature of the MOF@PES MMMs.

**Figure 5:**
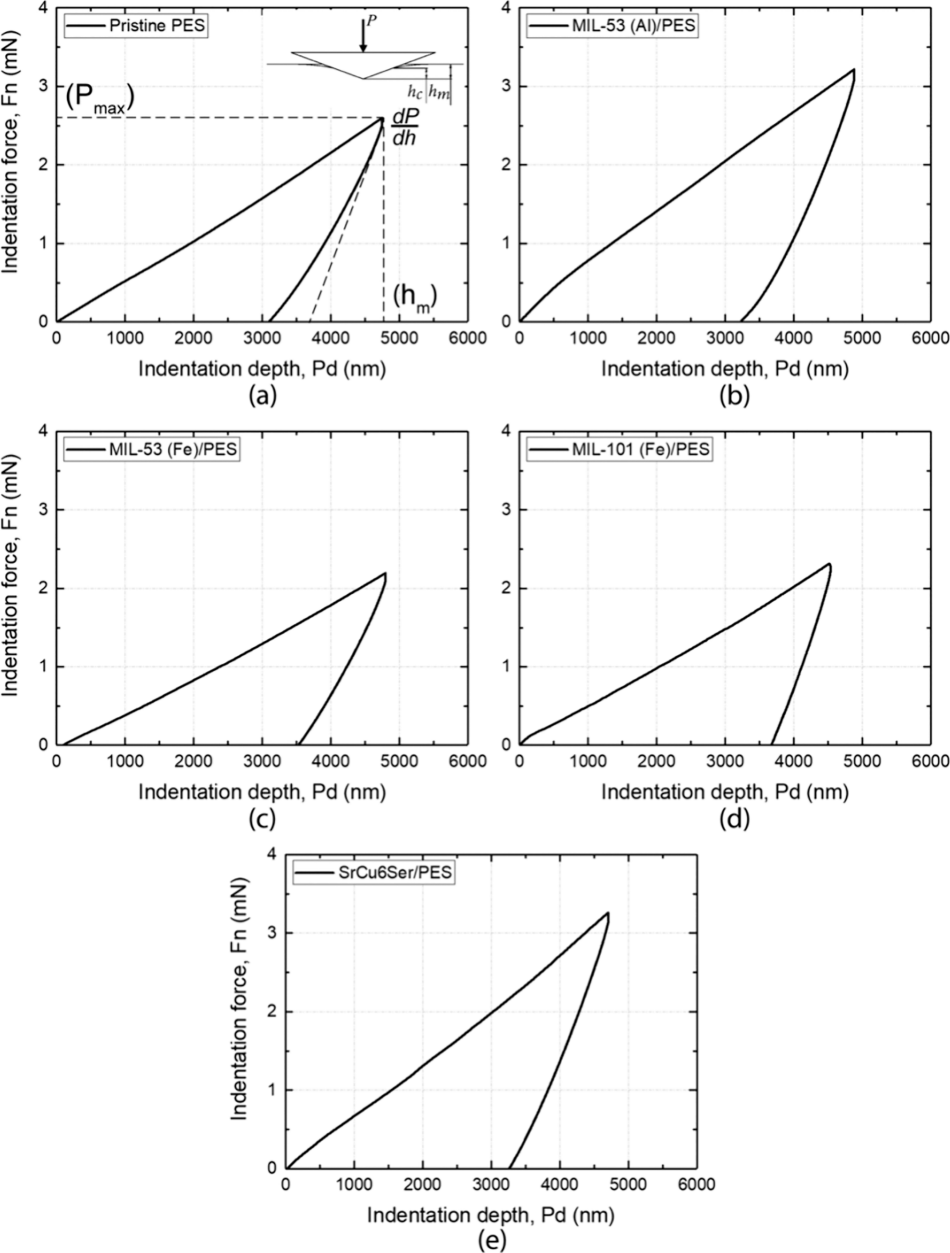
Force vs penetration depth (*F*_n_–*P*_d_) nanoindentation curves of all investigated materials: (a) **PES**, (b) **MIL-53(Al)@PES**, (c) **MIL-53(Fe)@PES**, (d) **MIL-101(Fe)@PES**, and (e) **SrCu**_**6**_**Ser@PES**.

Remarkably, in terms of Young’s modulus (*E*) and yield strength (σ_*y*_), MOF@PES MMMs exhibited better performance than the PES membrane (Figure S9). This could be understood as a direct consequence of the higher stiffness of the MOF fillers with respect to the PES polymer matrix. On the other hand, mechanical properties at the micronano scale, measured by instrumented nanoindentation tests, provide useful insights into estimating the mechanical response of membranes subjected to localized contact forces, possibly caused by interactions with sharp objects. These measurements evidenced a similar trend between global (*E*) and local stiffness values (obtained from Young’s indentation modulus, *E*_IT_) (Figure 5 and Figure S10). So, MOF@PES MMMs exhibit a better performance than the PES membrane samples, with indentation modulus ranging from 85.24 ± 10.55 MPa (**MIL-53(Fe)@PES**) to 114.19 ± 11.65 MPa (**MIL-101(Fe)@PES**), which is directly related to the increase of the contact stiffness due to the presence of the MOF as filler. The higher variability of local results with respect to the global ones are most likely attributed to the nonhomogeneous structure of the materials. **SrCu**_**6**_**Ser@PES** is the material with the smallest mismatch between both scales (*E*_IT_ = 105.2 ± 16.7 MPa and *E* = 112.4 ± 9.4 MPa). This variability also occurs for indentation hardness (*H*_IT_) spanning from 4.69 ± 0.26 to 7.42 ± 0.92 MPa for **MIL53-(Fe)@PES** and **SrCu**_**6**_**Ser@PES**, respectively.

### MOF Adsorption Performances for Nickel(II) and Cobalt(II) Cations

**Figure 6:**
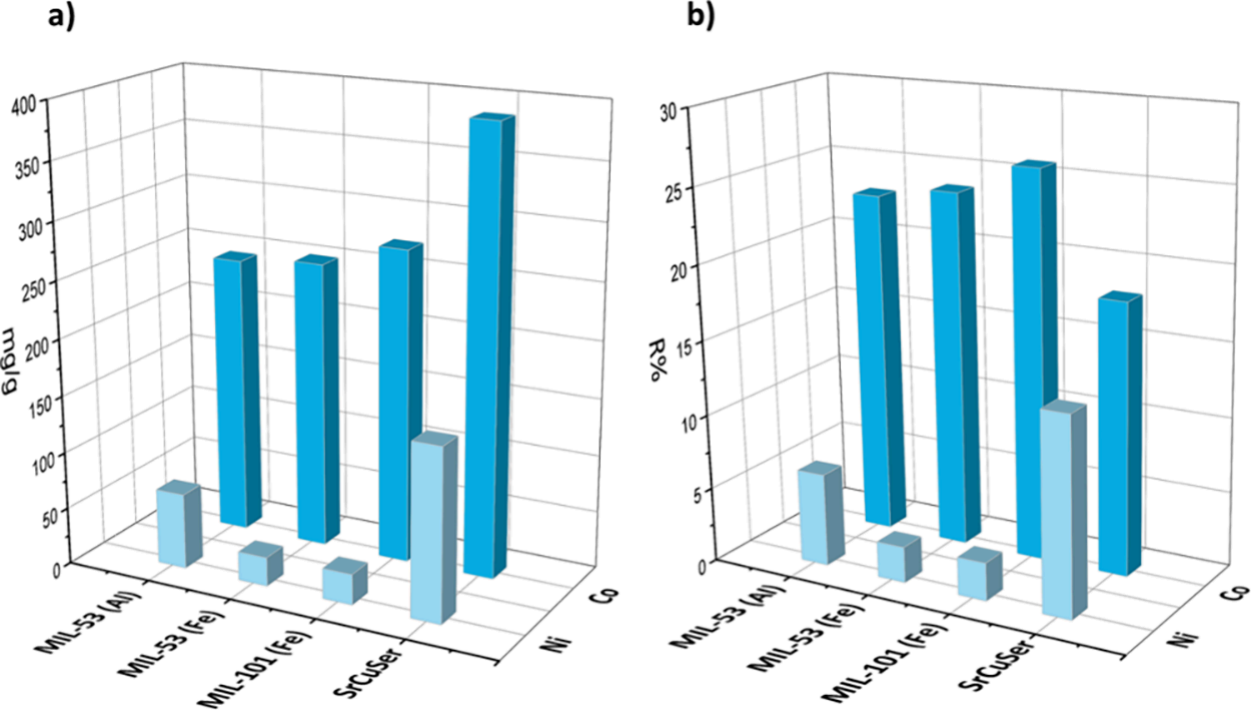
Equilibrium maximum loading (a) and removal efficiency (b) determined by soaking 20 mg of polycrystalline samples of the selected MOFs (from the left: **MIL-53(Al)**, **MIL-53(Fe)**, **MIL-101(Fe)**, and **SrCu**_**6**_**Ser**) in a 10 mL aqueous solution containing the suitable metal salt (1000 mg/g of Ni(NO_3_)_2_ (a) or 2000 mg/g of Co(NO_3_)_2_ (b)) (graphics are organized from data reported in Table S1).

Prior to testing the MOF@PES MMMs, we studied the performance of MOFs as polycrystalline powders for the single removal of nickel(II) and cobalt(II) cations from water. We soaked 20 mg of each MOF for 72 h in 10 mL of an oligomineral aqueous solution containing either Ni(NO_3_)_2_ or Co(NO_3_)_2_ metal salt at a concentration of 1000 mg/g (2000 mg/L) and 2000 mg/g (4000 mg/L), respectively. Then, small aliquots of sample were extracted after 3 days, and the metal ratio was monitored through ICP-MS, allowing the equilibrium maximum loading (Qe) and the removal efficiency (*R*) of each MOF to be obtained (Figure 6).

All of the MOFs showed comparable performances for cobalt(II) ion removal, with **SrCu**_**6**_**Ser** showing the best results, with the highest Qe of 387.3 mg/g, which can be attributed to the ideal combination of pore size and functionality, determined by OH-functionalized arms of serine residues. On the contrary, the removal of nickel(II) was lower for all the MOFs with **SrCu**_**6**_**Ser** still showing the best performances, with a Qe of 150.8 mg/g. These results suggest that, at least when a high concentration of metal ions is used, all the MOFs are good candidates for Co(II) and, to a lower extent, for Ni(II) removal, with **SrCu**_**6**_**Ser** being the best adsorbent for both metal ions, most likely due to the optimal combination of pore size and environment and also high crystallinity of the phase.

### PES Membrane and MOF@PES MMM Adsorption Performances for Nickel(II) Cations

We performed a preliminary evaluation of the capture properties of neat PES membranes and MMMs-MOFs for Ni(II) ions in batch experiments. Ni(II) metal ion was chosen for this preliminary screening to assess if MOFs embedded in MMMs preserved their porosity and adsorption ability toward this metal ion, which showed a reduced affinity for the MOFs in the maximum loading experiments. Moreover, we were able to determine a working concentration range, wherein MOFs showed maximum removal efficiency for the recovery of nickel(II). In this study, we used 100 mL of ordinary oligo-mineral water (see composition in Table S2), to which a known concentration (1 ppm) of Ni(NO_3_)_2_ was added to study the effect of other interfering cations. The variation of Ni(II) ion concentration was monitored at regular intervals of time (Table S3). The initial variation of the concentration fluctuates, most likely due to adsorption–desorption processes, until reaching an equilibrium, when the concentration gradually decreases upon time (Figure S11).

**Figure 7:**
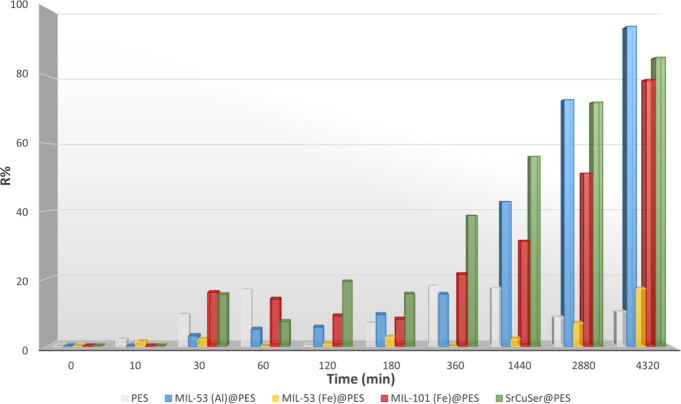
Removal efficiency for Ni(II) ion of the neat PES membrane and MOF@PES MMMs vs time. Graphics are organized from data reported in Table S3.

As expected, a very low removal efficiency was obtained with the PES membrane (*R* = 10.2%) (Figure 7). The pores of the membrane are too large with respect to the ionic radius of the metal ion for retaining it, and the membrane does not possess suitable functional groups able to strongly interact and adsorb Ni(II) species.^68,69^

The maximum removal efficiency for Ni(II) ion was achieved by **MIL-53(Al)@PES** (*R* = 94.6%) followed by **SrCu**_**6**_**Ser@PES** (*R* = 85.5%) and **MIL-101(Fe)@PES** (*R* = 78.9%). A very low removal efficiency (*R* = 17.1%) was obtained for **MIL-53(Fe)@PES** with respect to those of the other MOFs. The ability of **MIL-53(Al)@PES** to efficiently remove nickel(II) metal ions can be attributed to the high porous surface and free pore volume of this MOF, as well as its suitable pore dimensions (8.5 × 8.5 Å). Furthermore, **MIL-53(Al)** presents a flexible framework structure able to adapt for guesting ions and small molecular species. In addition, the notable removal efficiency of **SrCu**_**6**_**Ser@PES** is most likely related with the medium-sized (∼9 Å) functional hexagonal channels decorated with hydroxyl (−OH) groups from the −CH_2_OH serine amino acid residues, which can establish suitable interactions with Ni(II) ions. In turn, the **MIL-101(Fe)** structure encompasses two different mesoporous cages: small cages with pentagonal windows and large cages with pentagonal and hexagonal windows, with diameters of 29 and 34 Å, respectively. So, taking into account the van der Waals radii of the framework walls and average pore apertures of 12 and 16 Å, the cage dimensions are greater than the metal ion radius, which can explain the lower efficiency of **MIL-101(Fe)@PES**. Moreover, because both Fe(II) and Fe(III) can be present in the framework structure of both **MIL-53(Fe)** and **MIL-101(Fe)**, some extra-framework iron ions can be included into the pores to balance the overall negative charge of the 3D network, thus reducing the nominal high pore surface of the MOF.^70^

The values of the equilibrium maximum loading (Qe) for the neat PES membrane, **MIL-53(Al)@PES**, **MIL-53(Fe)@PES**, **MIL-101(Fe)@PES**, and **SrCu**_**6**_**Ser@PES** were 366.0, 3188.0, 502.0 1634.0 and 1195.1 μg/g, respectively (Table S3).

The concentration of common metal cations present in the oligo-mineral water was also monitored over time for **MIL-53(Al)@PES** (Figure S12). No appreciable variation of the concentration of common metal ions was observed, demonstrating that they are not competitive toward Ni(II) adsorption. A similar behavior can be reasonably assumed for all the other MOF@MMMs due to the low propension of these metal to coordination.

### PES Membrane and MOF@PES MMM Adsorption Performance for the Dual Capture of Ni(II) and Co(II) Metal Cations

**Figure 8:**
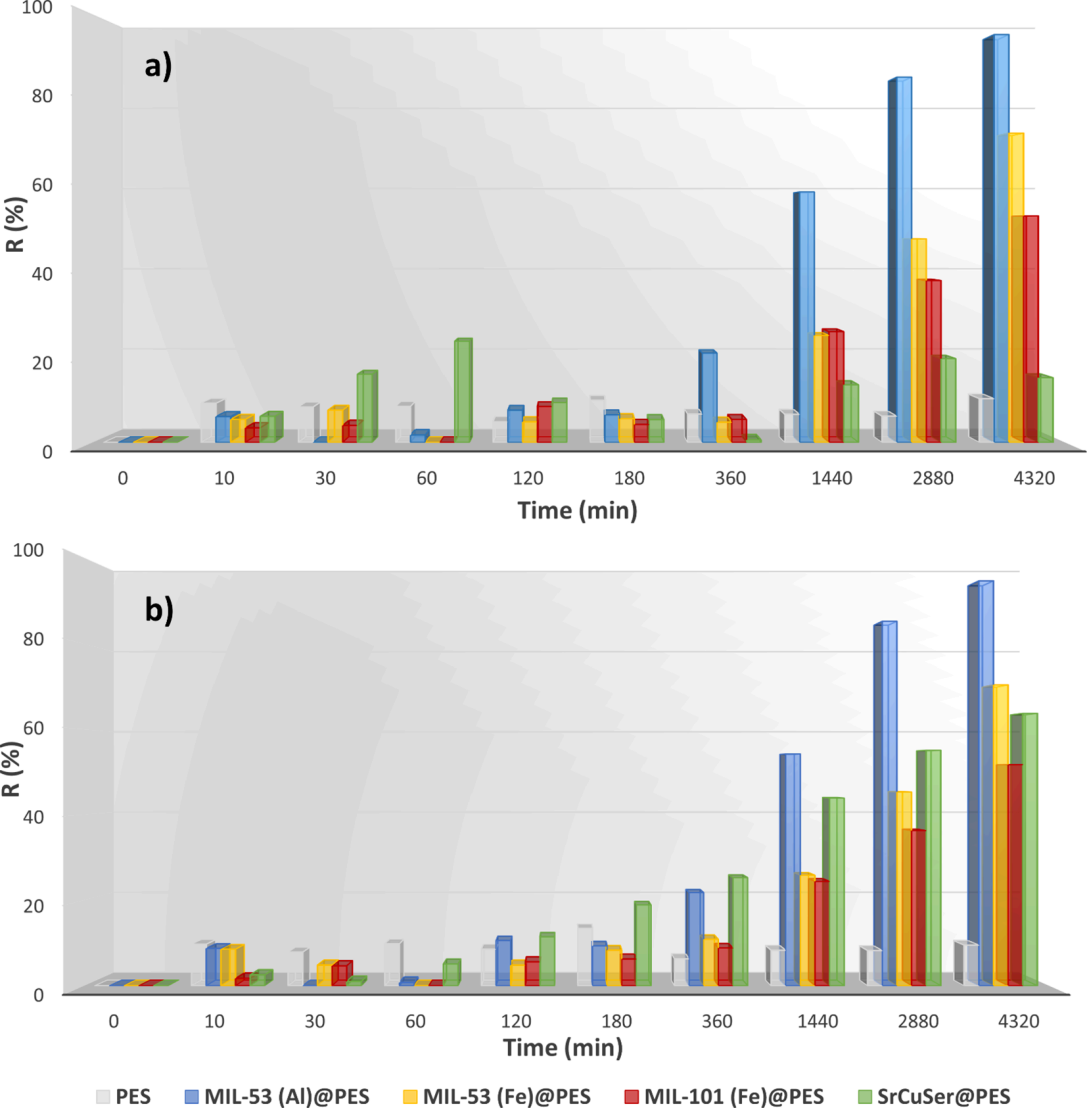
Removal efficiency toward (a) Ni(II) and (b) Co(II) ions determined by soaking MOF@PES MMMs in 100 mL of oligo-mineral aqueous solution containing 1 ppm of Ni(NO_3_)_2_ and 5 ppm of Co(NO_3_)_2_. Graphics are organized from data reported in Table S5.

Because a variety of heavy metal ions can be simultaneously present in wastewater, a material that can simultaneously adsorb different species can be useful for some practical application, such as water remediation. Conversely, when thinking of the recovery of strategic metal ions due to their technologic and economic relevance, their selective adsorption represents a necessary target. However, this is a very challenging task due to metal ion similarities in terms of size and/or binding affinities toward active functional groups, which eventually preclude the preferential adsorption of certain species with respect to others. Nickel and cobalt are commonly found in Li-ion battery waste together with other metal ions, such as copper, manganese, and lithium (Table S4). They have comparable ionic radius (5.8–6.5 Å for Co(II) and 5.5–6.9 for Ni(II) Å, depending on its spin state), and in water, they are usually present as hexacoordinated water complexes. They feature similar chemical properties and comparable affinity toward hard coordination sites. Consequently, it is not straightforward to separate these metal ions because of their size or binding preference. In this context, we studied the dual removal efficiency of the prepared PES membrane and MOFs@PES MMMs for cobalt(II) and nickel(II) ions using an ordinary oligo-mineral water (see composition reported in Table S2) to which known concentrations of Ni(NO_3_)_2_ (1 ppm) and Co(NO_3_)_2_ (5 ppm) were added. From these experiments, we observed that the PES membrane slightly contributes to the adsorption of metal ions (*R* of 10.2% and 9.5% for Ni(II) and Co(II), respectively), confirming what was already observed during the absorption experiments for the sole Ni(II). The amount of both metal ions removed by **MIL-53(Al)@PES** was higher than the amount adsorbed by the other membranes in the same conditions, as reported in Figure 8, Figure S13, and Table 1 (*R* = 94.7 and 95.4% for Co(II) and Ni(II), respectively). In turn, the isoreticular **MIL-53(Fe)@PES** again provided lower removal efficiencies, 70.4 and 72.4% for Co(II) and Ni(II), respectively, which somehow further corroborate the previous hypothesis that extra-framework iron atoms can be present in the MOF pores and consequently reduce the accessible free pore volume. Nevertheless, the capability of this MOF to capture nickel(II) ions increases in the presence of cobalt(II), most likely caused by coadsorption phenomena. **MIL-101(Fe)@PES** showed even lower performance, 51.7 and 53.0% for Co(II) and Ni(II), respectively, confirming a reduced ability of this MOF for nickel and cobalt removal.

**Table 1:** Equilibrium Loading (Qe) for Each Membrane Obtained by Soaking the MOF@PES MMMs in a 100 mL of Oligo-Mineral Aqueous Solution Containing 1 ppm of Ni(NO_3_)_2_ and 5 ppm of Co(NO_3_)_2_

	**Qe** (μg/g) for Co(II)	**Qe** (μg/g) for Ni(II)
**PES**	1123.5	321.3
**MIL-53(Al)@PES**	9735.6	2550.6
**MIL-53(Fe)@PES**	4835.6	1297.4
**MIL-101(Fe)@PES**	3067.9	7839.1
**SrCu**_**6**_**Ser@PES**	4426.0	275.3

Surprisingly, **SrCu**_**6**_**Ser@PES** (BioMOF) showed a higher removal efficiency for Co(II) (63.7%) (Figure 8b) with respect to Ni(II) (15.1%) (Figure 8a), evidencing a noteworthy selectivity, which most likely relies on the nature of MOF channels in terms of size, shape, and hydroxyl functionality.

Noteworthily, based on their adsorption capacity and the already mentioned results obtained for **MIL-53(Al)@PES** (Figure S12), the presence of other common ions in oligo-mineral water does not interfere with the ability of the MOF@PES MMMs in removing targeted metal ions from water even though there are much higher concentration values of Na(I) with respect to the targeted metal ions, evidencing that the ability of the composite materials to selectively recognize and remove the heavy metal ions from water is preserved even in the presence of other common cations.

The equilibrium loading (Table 1) for neat PES membrane and MOF@PES MMMs, achieved after 3 days, supports the previous results, situating **MIL-53(Al)@PES** as the best adsorbent for the simultaneous removal of nickel(II) and cobalt(II) heavy metal ions from water and validating **SrCu**_**6**_**Ser@PES** as a potential effective material for the selective capture of Co(II) from water, allowing the separation of these two very similar metal ions from solution.

**Figure 9:**
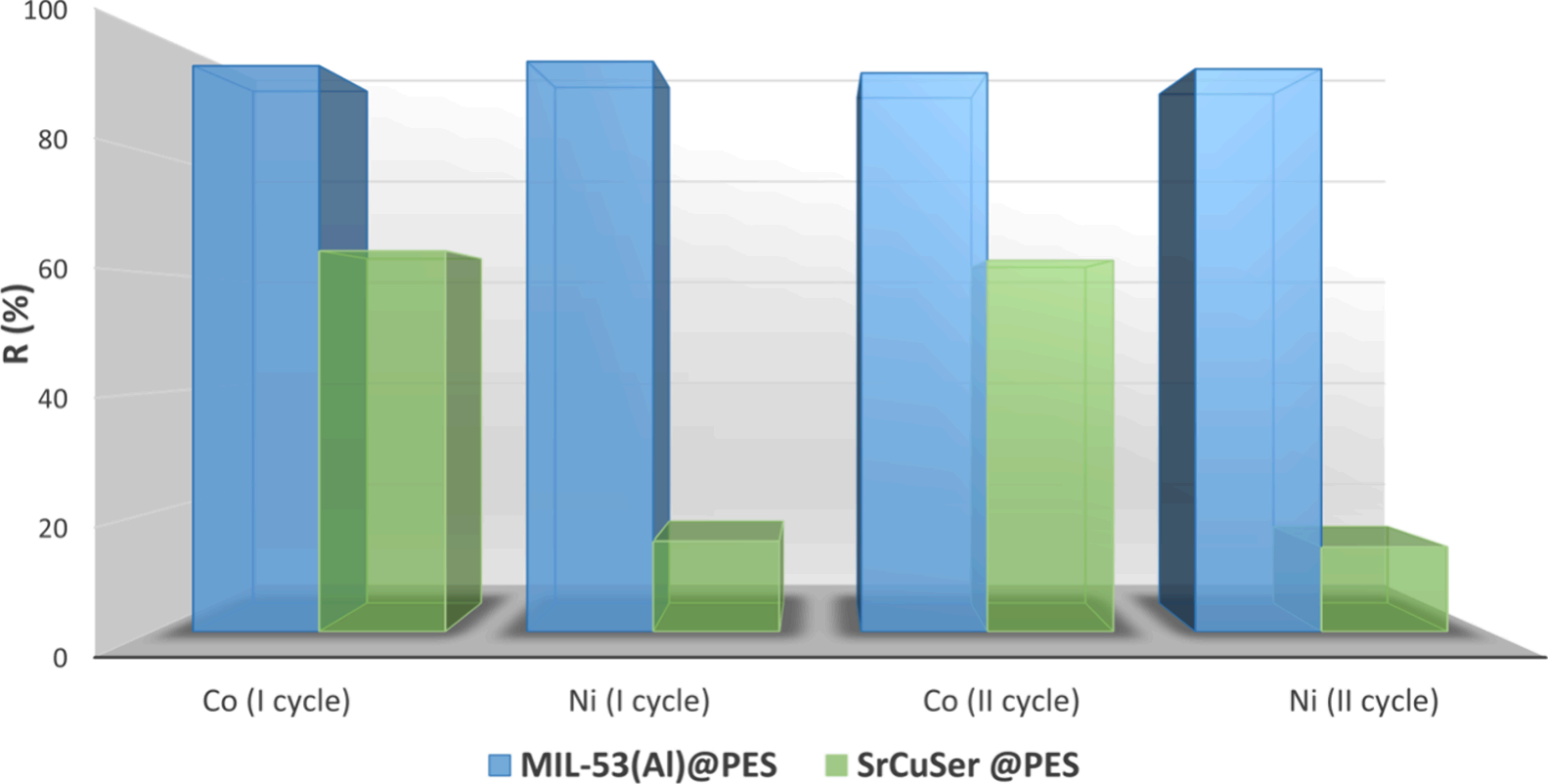
Removal efficiency toward (a) Ni(II) and (b) Co(II) cations determined by soaking **MIL-53(Al)@PES** and **SrCu6Ser@PES** in 100 mL of oligo-mineral aqueous solution containing 1 ppm of Ni(NO_3_)_2_ and 5 ppm of Co(NO_3_)_2_). Graphics are organized from data reported in Table S6.

To gain some insight into the spatial distribution and nature of adsorbed metal ions, we have performed SEM-energy dispersive X-ray spectroscopy (SEM-EDX) and X-ray photoelectron spectroscopy (XPS) on MOFs@PES after dual capture experiments. SEM-EDX elemental mapping allowed to observe a homogeneous distribution of nickel(II) and cobalt(II) metal ions on the MMMs (Figure S14). On the other side, XPS experiments have been carried out for **SrCu**_**6**_**Ser@PES** as a representative example. From it, we extracted that the oxidation state of nickel(II) and cobalt(II) metal ions was retained (Figure S15). Thus, Co 2p XPS spectra are shown in Figure S15a. Both main peaks of Co 2p_3/2_ and Co 2p_1/2_, with binding energies of 781.4 and 787.3 eV, respectively, are indicative of Co^2+^.^71^Figure S15a also shows the presence of the two characteristic satellites centered at 786.2 and 802.8 eV. On the other side, the Ni 2p XPS spectra (Figure S15b) are also characteristic of a +2 oxidation state.^72^Figure S15b shows two main peaks corresponding to Ni 2p_3/2_ and Ni 2p_1/2_, centered at 855.9 and 872.8 eV, respectively, accompanied with satellite peaks at around 861 and 878 eV. Finally, because membrane stability and regeneration are key factors for their industrial or other real-world applications, the stability of all MOF@PES MMMs was confirmed by PXRD after use (Figure S16), and their reusability was tested with **MIL-53(Al)@PES** and **SrCu**_**6**_**Ser@PES**, evidencing the robustness of both membranes during the regeneration process. The MOF@PES MMMs were then regenerated by soaking in a 4:1 water/ethanol mixture for 24 h and then reused for capture experiments. After two cycles, a minor decline of their initial performance was observed, as shown in Figure 9, with both MOF@PES MMMs still showing significant efficiency recovery after each regeneration process, losing less than 5% of the original adsorption capacities (Table S6 and Figure 9).

## Conclusions

In summary, we have investigated the efficiency and selectivity of a new series of mixed matrix membranes based on metal–organic frameworks (MOF@PES MMMs) in the recovery of cobalt(II) and nickel(II) from aqueous solutions. Specifically, we synthesized and characterized four poly(ether sulfone) (PES)-based MMM-MOFs with four previously reported MOFs, i.e., **MIL-53(Al)**, **MIL-53(Fe)**, **MIL-101(Fe)**, and **SrCu**_**6**_**Ser**, chosen for their unique characteristics such as permanent porosity, large surface areas, ease of functionalization, controlled surface charges, and high water stability. The resulting MOF@PES MMMs exhibit remarkable capture efficiencies for cobalt and nickel even in the presence of common ions found in oligo-mineral water. These results represent one of the first examples of potential real-world application of MMM-MOFs, and moreover, it does so in a field as significant from both technological and environmental perspectives as the recovery of key metals in lithium-ion batteries.

## Experimental Section

### Physical Techniques

Elemental analyses (C, H, and N) were performed at the microanalysis service of the Dipartimento di Chimica e Tecnologie Chimiche of the Università della Calabria (Italy). FTIR spectra were recorded on a Nicolet-6700 spectrophotometer as KBr pellets. The thermogravimetric analysis was performed on crystalline samples under a dry N_2_ atmosphere with a Mettler Toledo TGA/STDA 851^e^ thermobalance operating at a heating rate of 10 °C min^–1^. The N_2_ adsorption–desorption isotherms at 77 K were carried out on polycrystalline samples of **MIL-53(Al)**, **MIL-53(Fe)**, and **MIL-101(Fe)** and (**SrCu**_**6**_**Ser**) and **MIL-53(Al)@PES**, **MIL-53(Fe)@PES**, **MIL-101(Fe)@PES**, and **SrCu**_**6**_**Ser@PES** with a BELSORP MINI X instrument. Samples were activated at 70 °C under reduced pressure (10^–6^ Torr) for 16 h prior to carrying out the sorption measurements.

#### Preparation of Polyether Sulfone (PES)

PES pellets (Aldrich 200 g, MW 58,000 g/mol) were heated at 110 °C for 24 h to remove humidity. Then, 25 wt % of PES solution was prepared by adding PES pellets (12.5 g, 0.21 mmol) to dimethyl sulfoxide (DMSO) (37.5 g, 479.96 mmol), and the solution was kept under stirring in a sand bath heated at 60 °C. From the prepared solution, PES (1g, 0.017 mmol) was added to a vial containing DMSO (1g, 12.79 mmol) to obtain a 12.5 wt.% solution. The vial was kept under stirring in a sand bath heated at 65 °C. Following this step, the prepared solution underwent ultrasonication to remove eventual air bubbles and to ensure the homogeneity of the solution.

#### Preparation of **MIL-53(Al)**

The present study employed the widely used solvothermal synthesis process for preparation of **MIL-53(Al)**.^26,73^ Solution A was prepared by weighing aluminum(III) nitrate nonahydrate (Al(NO_3_)_3_·9H_2_O) (98% purity) (1.12 g, 2.98 mmol) and placing it in 15 mL of *N*,*N*-dimethylformamide (DMF). Solution B was done by adding 1,4-benzenedicarboxylic (BDC) acid (98% purity) (1.11 g, 6.68 mmol) in 15 mL DMF. Each solution was left under continuous stirring for 1 h. Afterward, metal solution was added dropwise to the ligand, and the resulting solution was left under stirring at 300 rpm for 20 min at room temperature (RT). The mixture was then transferred to a 56 mL Teflon-lined hydrothermal autoclave. Finally, an oven was used to heat the sample to 120 °C for 72 h. Subsequently, the Teflon-lined autoclave was gradually cooled down to room temperature. Then, the solid phase was separated using centrifugation (HERMLE) at 6000 rpm and was washed three times with DMF and MeOH to remove the unreacted terephthalic acids trapped in the pores of the obtained product. The MOF was dried at RT. The product was a solid white powder, which was activated at 70 °C for 12 h and was subjected to different analyses for identifying and ensuring the correct formation of **MIL-53(Al)**.

#### Preparation of **MIL-53(Fe)**

The solvothermal synthesis of **MIL-53(Fe)** was conducted according to the literature^74^ but with minor modifications compared to the previous preparation methods. FeCl_3_ (0.54 g, 3.32 mmol) and H_2_BDC (0.33 g, 1.98 mmol) were added to separate beakers containing 15 mL of DMF, and the solutions were kept under continuous stirring at 300 rpm until complete homogeneity. Afterward, the metal solution was added drop-wisely to the ligand solution, and the final solution was kept under stirring at 300 rpm for 15 min. This slurry was then transferred to a 56 mL Teflon-lined stainless-steel autoclave and heated in an oven at 150 °C for 24 h. After cooling down, particles were obtained by centrifugation (HERMLE) at 6000 rpm, washed with fresh DMF and ethanol, and finally dried in the oven at 80 °C for 24 h. The product was gradually cooled down to RT. In the final step, a yellow brown powder product was obtained.

#### Preparation of **MIL-101(Fe)**

The following solvothermal synthesis has been modified from a previously published method.^75^ FeCl_3_ (0.67 g, 4.13 mmol) and terephthalic acid (0.205 g, 1.24 mmol) were put in two different beakers containing 8 mL of DMF which were kept under stirring at 300 rpm for 15 min. Afterward, the dissolved metal solution was added drop by drop to the ligand solution. Furthermore, it was left under stirring at 300 rpm for 15 min. This solution was put in a 56 mL Teflon-lined stainless-steel autoclaves and heated at 110 °C for 20 h. After cooling the reaction to RT, the desired product was isolated via centrifugation and was washed with DMF and MeOH. Then, it was dried at 80 °C for 12 h. Finally, the product was gradually cooled down. A dark brown product was activated at 150 °C for 10 h and was stored for characterization.

#### Preparation of {SrCu_6_[(*S*,*S*)-serimox]_3_(OH)_2_(H_2_O)}·39H_2_O (**SrCu_6_Ser**)

A multigram-scale procedure was carried out adapting a reported procedure^29^ by first preparing separately (Me_4_N)_2_{Cu_2_[(*S*,*S*)serimox] (OH)_2_}·5H_2_O (2.06 g, 3.49 mmol) and Sr(NO_3_)_2_ (0.25 g, 1.18 mmol) solutions in basic water at pH = 11.31 (10 and 6 mL, respectively) and then, under stirring, adding dropwise the salt solution on the other one, obtaining a turquoise precipitate that was stirred overnight. The precipitate was separated from the solution by centrifugation, and then it was washed with H_2_O (25 mL, ×3) and MeOH (25 mL, ×3). Finally, the turquoise solid was dried under vacuum conditions.

#### Preparation of MOF@PES MMMs

Different MOF@PES composites of **MIL-53(Al)**, **MIL-53(Fe)**, **MIL-101(Fe)**, and **SrCu**_**6**_**Ser** were prepared. All the MOFs were first activated before membrane preparation. Different MOF powders (11.11 wt %) based on the total mass mixture were added to small vials. In each vial, DMSO (1 g, 12.79 mmol) was added to 0.25 g of each MOF and kept under mechanical stirring at RT. The suspension was left until homogeneity. Afterward, 25 wt % PES stock solution (1 g, 0.017 mmol) was gradually added to the previously prepared MOF solution and was kept under stirring in a heated sand bath.

The membranes were casted using an Automatic Film Applicator. The casting rate was applied at 25 mm/s with a casting distance of 280 mm. A 250 μm thickness casting knife was used. Finally, the solutions were poured on a glass plate, and then the membranes were formed by a nonsolvent immersion precipitation separation method (NIPS). The casted membranes were immersed into a deionized water bath for 24 h and washed several times. Finally, the membranes were removed from the coagulation bath, stored on cleaned tissue paper, and left to dry at RT overnight. The membranes were stored for characterization.

#### Capture Experiments

Selected salts (nickel nitrate hexahydrate and cobalt nitrate hexahydrate) in this study were obtained from Honeywell (Charlotte, North Carolina, US). Ultrapure water was obtained from a Milli-Q plus system (Millipore, Bedford, MA). Individual standard solutions (at 1 and 5 mg L^–1^) of nickel nitrate hexahydrate and cobalt nitrate hexahydrate were respectively prepared in commercial mineral water (FIORDACQUA, Italy). MOF@PES MMMs were soaked in heavy metal ions solutions. Samples (1 mL) were analyzed at regular intervals of time (0, 10, 30, 60, 120, 180, 360, 1440, 2880, and 4320 min), and they were stabilized with 100 μL of nitric acid and 9 mL of Milli-Q water. To determine the captured metals by the membranes at each time, inductively coupled plasma-mass spectrometry (ICP-MS) was used.

#### Maximum Loading Experiments

To determine the maximum loading capacity, polycrystalline samples of each MOF (20 mg) were immersed into two 1000 mg/g solutions (10 mL) of nickel nitrate hexahydrate and 2000 mg/g solutions (10 mL) of cobalt nitrate hexahydrate. Samples were taken (1 mL) at 0, 1440, 2880, and 4320 min, and they were stabilized with 100 μL of nitric acid and 9 mL of Milli-Q water. ICP-MS was used to analyze each sample solution.

#### X-ray Powder and Membrane Diffraction Measurements

Fresh polycrystalline samples of each MOF and MOF@PES MMMs were mounted and aligned on a diffractometer (Bruker, D2 PHASER second generation, Germany) with Cu Kα radiation and a wavelength of 1.54056 Å. Measurements were collected at room temperature (2θ = 5–40°).

#### Macromechanical Tests

Rectangular-shaped samples (8 × 50 mm) were used for mechanical tensile tests at the macroscale. Samples were cut along the casting direction, and three replicates were analyzed for each material type. Displacement controlled tests (*ḋ* = 1 mm/min) were carried out using a universal tensile testing machine (MTS, Criterion C42, max load 100 N). Young modulus (*E*), yield strength (σ_y_), ultimate tensile strength (σ_ut_), and elongation to fracture (ε_f_) were directly obtained from the measured engineering stress–strain (σ–ε) curve. Stress values were obtained as the ratio between the tensile force and initial cross-sectional area of the sample. Strain quantities were the ratio between sample elongation and the initial length. Full field displacement/strain measurements were also carried out by an *in situ* digital image correlation (DIC) method. A high-resolution digital camera (Sony ICX 625-Prosilica GT 2450 model, 2448 × 2050 pixels) equipped with suitable objective (Linos Photonics, Rodagon lens f. 80 mm) was used to focus the investigation area with a proper magnification (resolution of 230 pixels/mm). Correlation analyses were carried out by the DIC commercial software (Vic-2D, Correlated Solution) using a subset size of 51 pixels and a distance of 7 pixels between subset centers.

#### Nanomechanical Tests

Nanomechanical tests were carried out by a nanoindenter (NHT, CSM Instruments, Switzerland) equipped with a spherical diamond tip (*r* = 20 μm). Depth controlled tests were carried out (*ḋ* = 1000 nm/min) with a maximum penetration depth of 5000 nm and a dwell time of 5 s. Matrices of 5 × 5 indentations were made for each sample. Nanoindentation results were used to estimate the mechanical properties at the nanoscale in terms of nanohardness (*H*_IT_) and indentation Young’s modulus (*E*_IT_). Hardness (*H*_IT_) was determined as the ratio between the maximum indentation load *P*_max_ and the developed contact area *A*(*h*_c_) at penetration depth *h*_c_. The elastic modulus (*E*_IT_) was computed according to the Oliver and Pharr theory.^76^ In particular, the modulus was based on the contact stiffness (*S*), that is, the slope of the force–depth curve at the early stage of unloading (98–40% of the maximum load, *P*_max_).

## Supporting Information Available

Additional experimental details, including characterization images and metal recovery data; Tables S1–S6; and Figures S1–S16 (PDF)
