# Supplementary material for: Efficient Nickel and Cobalt Recovery by Metal–Organic Framework-Based Mixed Matrix Membranes (MMM-MOFs)
Source: ACS Sustain Chem Eng. 2024 Jul 31;12(32):12014–28. doi: 10.1021/acssuschemeng.4c03427 (PMC11323268; doi:10.1021/acssuschemeng.4c03427)
Supplement: Supplementary file 1 — sc4c03427_si_001.pdf [file sc4c03427_si_001.pdf]

Supporting Information for:

Efficient Nickel and Cobalt Recovery by Metal–Organic Framework-based Mixed Matrix Membranes (MMM-MOFs)

Amira Nour,[a] Waseem Iqbal,[a] Javier Navarro-Alapont,[b] Jesús Ferrando-Soria,[b] Pietro Magarò,[c] Rosangela Elliani,[a] Antonio Tagarelli,[a] Carmine Maletta,[c] Teresa F. Mastropietro,*[a] Emilio Pardo,*[b] Donatella Armentano*[a]

[a]Dipartimento di Chimica e Tecnologie Chimiche (CTC), Università della Calabria, Rende 87036, Italy.

[b]Instituto de Ciencia Molecular (ICMol), Universidad de Valencia, 46980 Valencia, Spain.

[c]Dipartimento di Ingegneria Meccanica, Energetica e Gestionale, Università della Calabria, Rende 87036, Cosenza, Italy.

Corresponding authors: Teresa Mastropietro (teresafina.mastropietro@unical.it), Emilio Pardo (emilio.pardo@uv.es) and Donatella Armentano (donatella.armentano@unical.it).

Number of Pages: 25

Number of Figures: 16

Number of Tables: 6

Physical Techniques. Elemental analyses (C, H, and N) were performed at the microanalysis service of the Dipartimento di Chimica e Tecnologie Chimiche of the Università della Calabria (Italy). FTIR spectra were recorded on a Nicolet-6700 spectrophotometer as KBr pellets. The thermogravimetric analysis was performed on crystalline samples under a dry N_2 atmosphere with a Mettler Toledo TGA/STDA 851e thermobalance operating at a heating rate of 10 ºC min^{-1}.

X-ray Powder Diffraction Measurements: Fresh polycrystalline samples of **MIL-53(Al)**, **MIL-53(Fe)**, and **MIL-101(Fe)**, pristine PES membrane and MOF@PES MMMs were deposited on a flat plate with a 5 cm diameter prior to being mounted on a Bruker D2 PHASER Diffraction System with Cu-Kα radiation (λ = 1.54056 Å). Five repeated measurements were collected at room temperature (2θ = 2–50) and merged in a single diffractogram.

Gas Sorption: The N_2 adsorption isotherms at 77 of samples of **MIL-53(Al)**, **MIL-53(Fe)**, **MIL-101(Fe)** and **SrCu$_6$Ser** were carried out on crystalline samples with a BELSORP MINI X instrument. Samples were activated at 70 °C under reduced pressure (10^{-6} Torr) for 16 h prior to carry out the sorption measurements. The Brunauer-Emmett-Teller (BET) surface areas were calculated from the N_2 adsorption isotherm according to the criteria reported by Rouquerol et al. and de Lange et al.[1]

Table S1. Equilibrium maximum loading and removal efficiency determined by soaking 20 mg of polycrystalline samples of the selected MOFs in a 10 mL aqueous solution containing the suitable metal salt (1000 mg/g of $Ni(NO_3)_2$ or 2000 mg/g of $Co(NO_3)_2$]. Graphics are reported in Figure 5.

	Ni(II)		Co(II)	
	mg/g	R(%)	mg/g	R(%)
**MIL-53(Al)**	66.3	6.2	245.4	23.0
**MIL-53(Fe)**	25.4	2.4	252.0	23.8
**MIL-101(Fe)**	26.8	2.5	274.4	25.9
**$SrCu_6Ser$**	150.8	13.2	387.6	18.1

Table S2. Composition of oligo mineral water used for capture experiments (initial concentration[a,b] of the interfering cations)

Compound	Concentration (μg/L)
Na^+	3369.2
K^+	1072.9
Mg^{2+}	929.7
Ca^{2+}	669.0

Table S3. Residual of Ni^{2+} concentration[a,b] in an oligo-mineral aqueous solution (volume 100 mL) containing $Ni(NO_3)_2$ at an initial concentration of *ca*. 1 ppm in presence of multi-ions as interfering media. Graphics are reported in Figure S6.

Time (min.)	**PES** (μg/L)	**MIL-53 (Al)@PES** (μg/L)	**MIL-53 (Fe)@PES** (μg/L)	**MIL-101 (Fe)@PES** (μg/L)	**$SrCu_6Ser$@PES** (μg/L)
0	1750	1771	1445	1636	1025.2
10	1714	1771	1425	1636	1025
30	1585	1600	1415	1374	868
60	1456	1569	1444	1406	950
120	1745	1559	1434	1489	829
180	1630	1497	1405	1504	866
360	1436	1396	1445	1286	629
1440	1449	945	1413	1127	449
2880	1600	447	1346	798	285
4320	1571	89.7	1198	345	149

[a]LOD: 0.015 ppb. [b]Each experiment was performed in triplicate and results are reported as average values ± 3 SD.

Table S4. Mean composition of Li-ion battery (smartphone battery).

Li-ion battery metals	mg/g
Li	38.4
Mn	63.5
Ni	70.0
Co	226
Cu	6.47

Table S5. Residual of Ni^{2+} and Co^{2+}concentration[a,b] in an oligo-mineral aqueous solution (volume 100 mL) containing $Ni(NO_3)_2$ at an initial concentration of *ca*. 1 ppb (top) and $Co(NO_3)_2$ at an initial concentration of *ca*. 5 ppb (bottom) in presence of multi-ions as interfering media. Graphics are reported in Figure S6.

Time (min)	**PES** (μg/L)	**MIL-53 (Al)@PES** (μg/L)	**MIL-53 (Fe)@PES** (μg/L)	**MIL-101 (Fe)@PES** (μg/L)	**$SrCu_6Ser$@PES** (μg/L)
0	1444	1283	1309	1398	1328
10	1312	1206	1238	1351	1247
30	1325	1283	1210	1343	1116
60	1323	1261	1309	1398	1014
120	1375	1187	1246	1281	1204
180	1302	1201	1236	1340	1257
360	1349	1015	1247	1323	1320
1440	1350	531	982	1036	1149
2880	1356	187	686	869	1068
4320	1297	60	362	657	1127

Time (min)	**PES** (μg/L)	**MIL-53 (Al)@PES** (μg/L)	**MIL-53 (Fe)@PES** (μg/L)	**MIL-101 (Fe)@PES** (μg/L)	**$SrCu_6Ser$@PES** (μg/L)
0	5433	4935	5016	5159	5071
10	4904	4503	4586	5070	4942
30	5005	4935	4772	4917	5019
60	4900	4896	5016	5159	4812
120	4967	4413	4773	4869	4490
180	4704	4474	4598	4835	4111
360	5091	3859	4470	4703	3787
1440	4981	2259	3719	3900	2845
2880	4986	728	2743	3286	2278
4320	4917	262	1486	2490	1840

[a]LOD: 0.015 ppb. [b]Each experiment was performed in triplicate and results are reported as average values ± 3 SD.

Table S6. Residual of Ni^{2+} and Co^{2+}concentration [a,b] in an oligo-mineral aqueous solution (volume 100 mL) containing $Ni(NO_3)_2$ at an initial concentration of *ca*. 1 ppb (top) and $Co(NO_3)_2$ at an initial concentration of *ca*. 5 ppb (bottom) in presence of multi-ions as interfering media after regeneration and reuse of the MOF@PES MMMs.

Time (min)	**MIL-53 (Al)@PES** (μg/L)	**$SrCu_6Ser$@PES** (μg/L)
0	1271	1355
10	1242	1284
30	1283	1149
60	1287	1044
120	1222	1241
180	1225	1295
360	1046	1360
1440	542	1183
2880	191	1100
4320	62	1166

Time (min)	**MIL-53 (Al)@PES** (μg/L)	**$SrCu_6Ser$@PES** (μg/L)
0	4886	5046
10	4660	5090
30	4688	5170
60	4651	4956
120	4501	4625
180	4563	4234
360	3975	3901
1440	2282	2930
2880	743	2346
4320	272	1895

[a]LOD: 0.015 ppb. [b]Each experiment was performed in triplicate and results are reported as average values ± 3 SD.

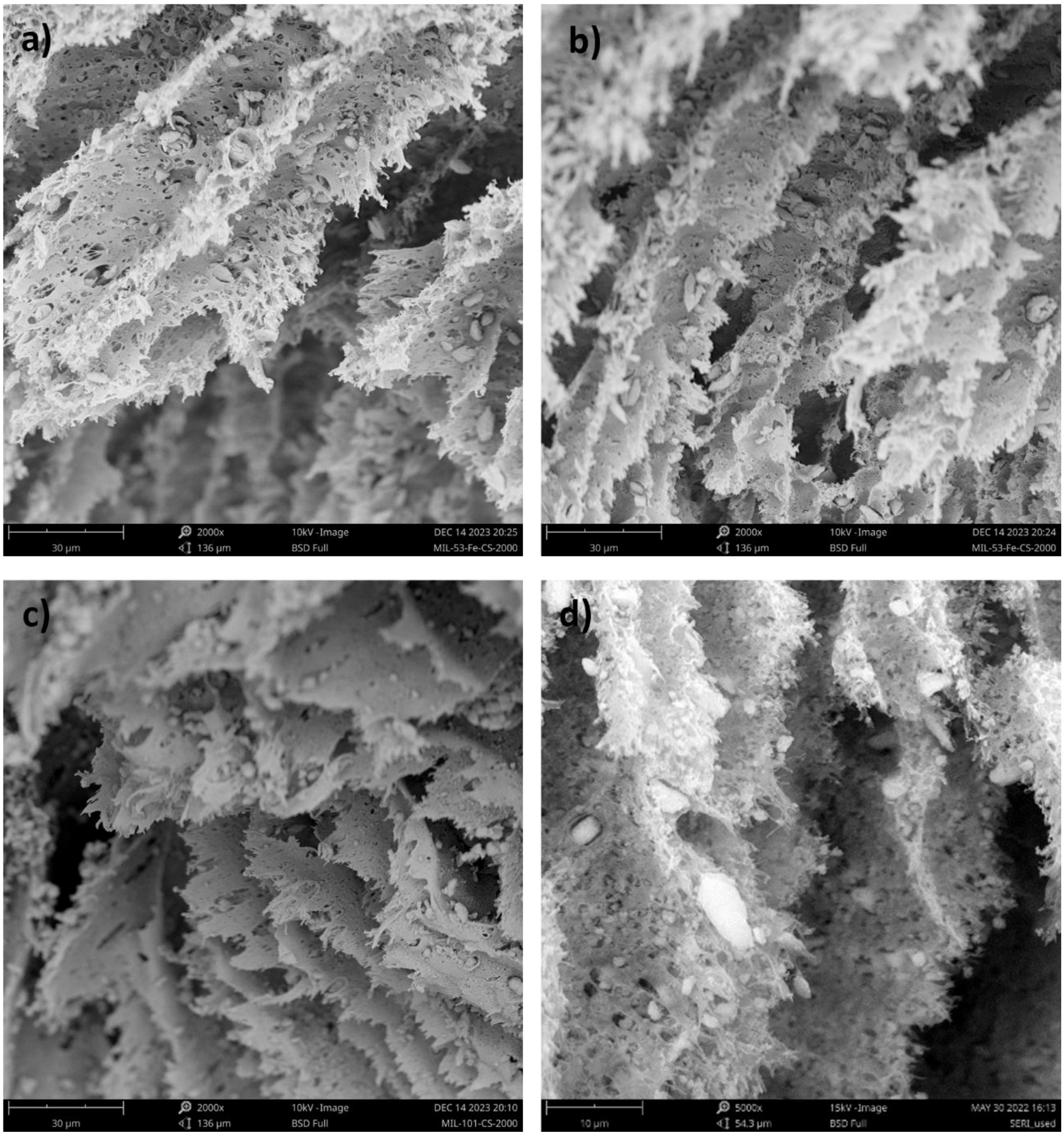

Figure S1. Scanning-electron microscopy of the cross-section **MIL-53(Al)@PES** (a) **MIL-53(Fe)@PES** (b), **MIL-101(Fe)@PES** (c) and **$SrCu_6Ser$@PES** (d).

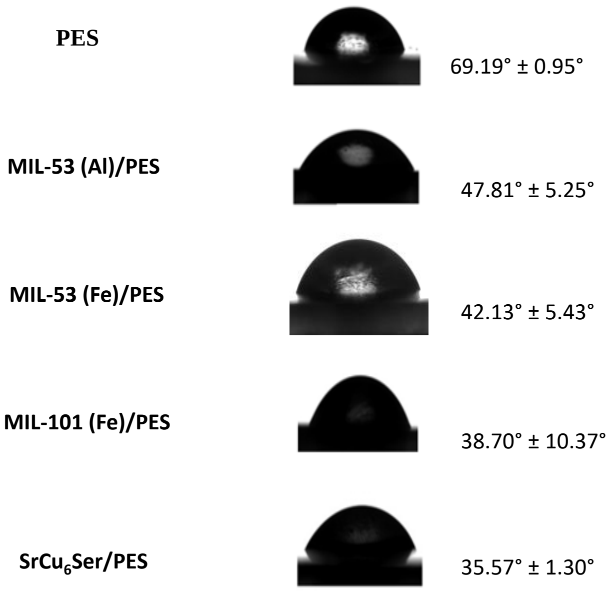

Figure S2. Water contact angle of the top surface of pristine PES and MOF@PES MMMs membrane.

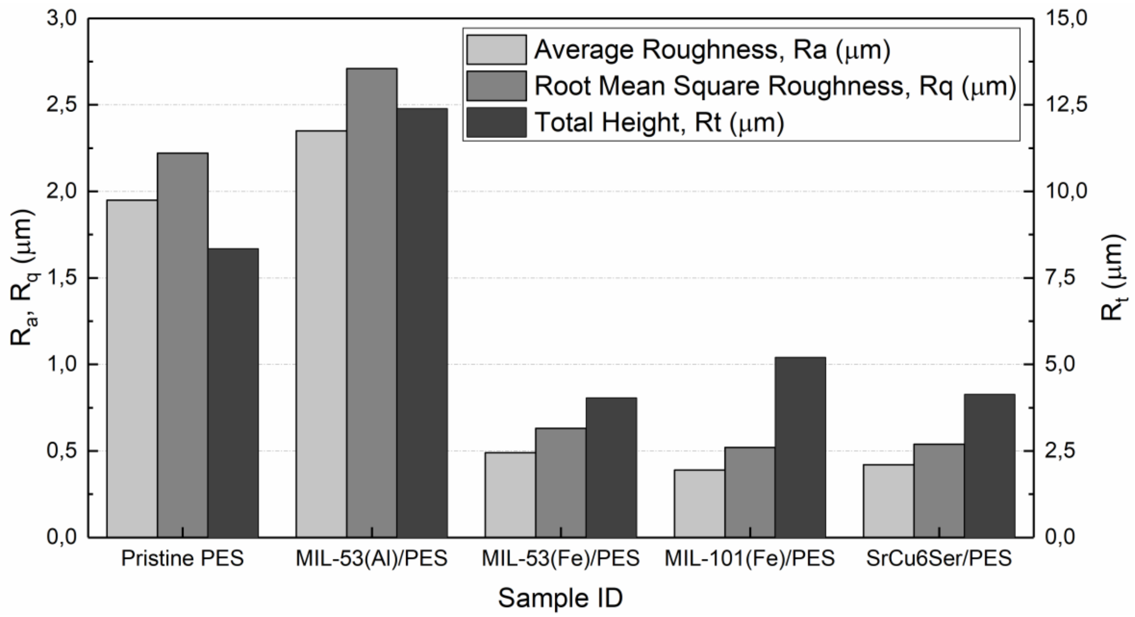

Figure S3. Roughness parameters (Ra, Rq, Rt) measured starting from profile scans carried out on MOF@PES MMMs.

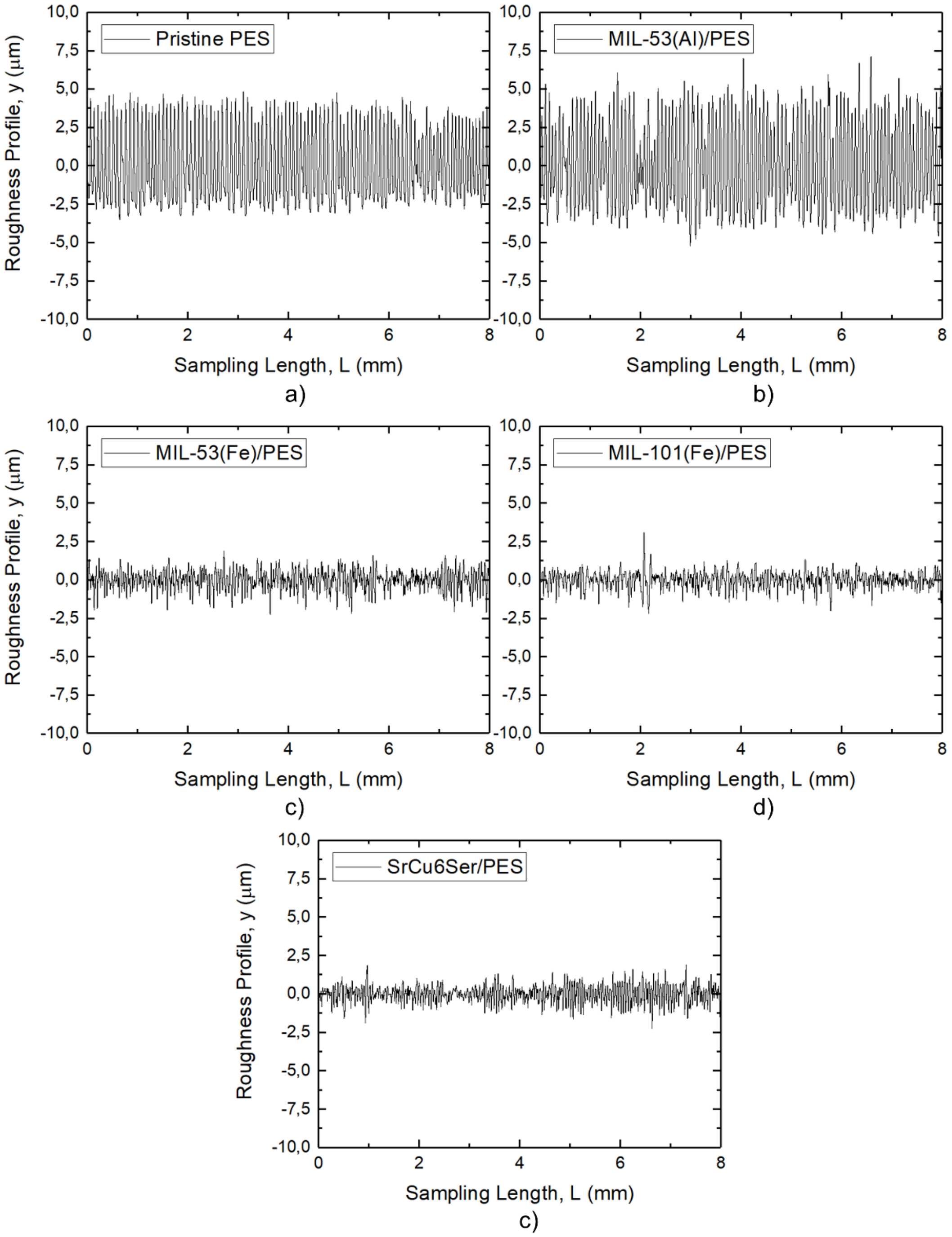

Figure S4. Roughness profiles of MOF@PES MMMs along a sampling length of 8 mm: a) Pristine PES, b) **MIL-53(Al)@PES**; c) **MIL-53(Fe)@PES**; d) **MIL-101(Fe)@PES** and e) **$SrCu_6Ser$@PES.**

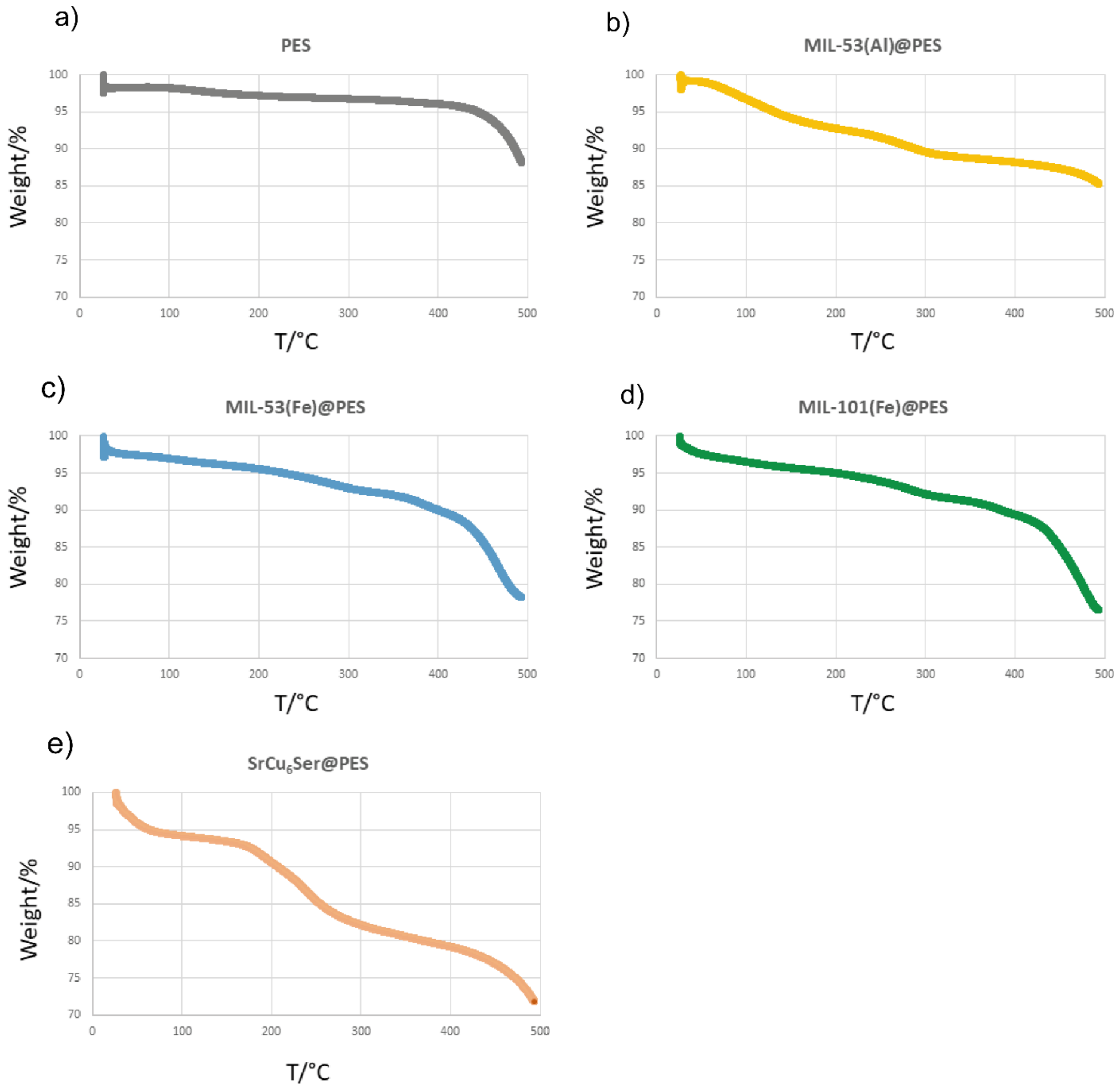

Figure S5. Thermo-Gravimetric Analysis (TGA) of a) **PES**; b) **MIL-53@PES(Al)**; c) **MIL-53(Fe)@PES**; d) **MIL-101(Fe)@PES**; e) **$SrCu_6Ser$@PES** e) under dry N_2 atmosphere.

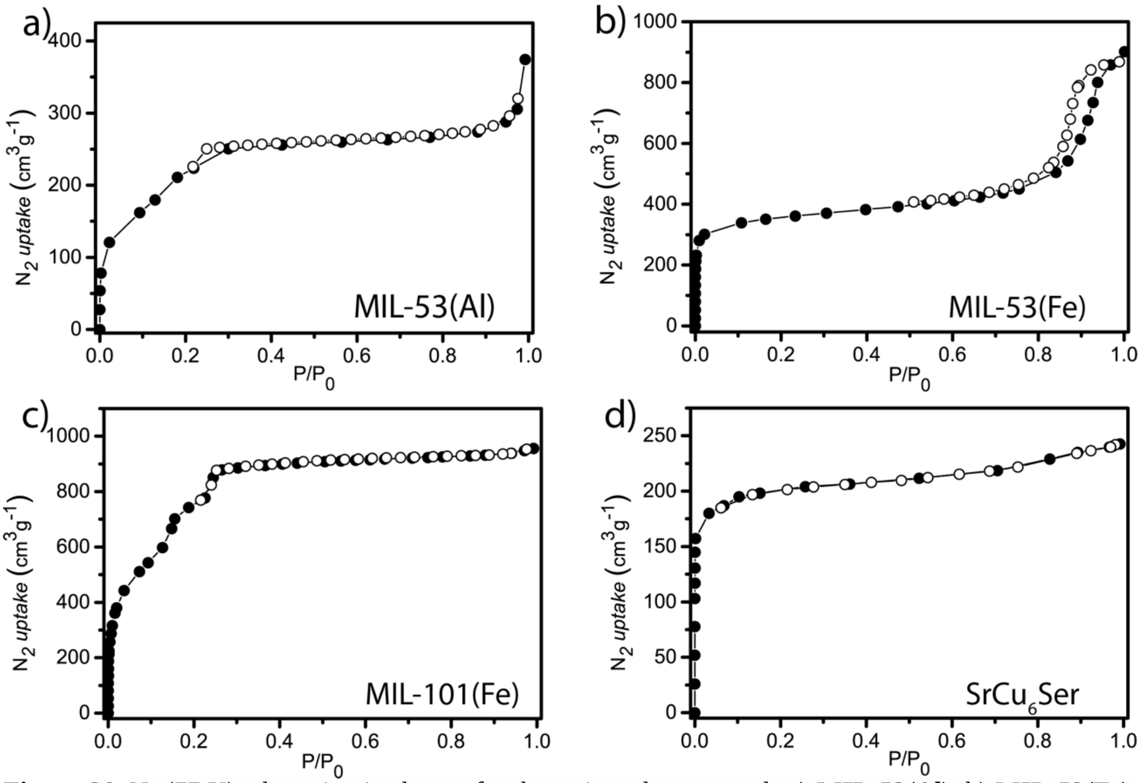

Figure S6. N_2 (77 K) adsorption isotherms for the activated compounds a) **MIL-53(Al)**; b) **MIL-53(Fe)**; c) **MIL-101(Fe)** and d) **SrCu₆Ser**. Filled and empty symbols indicate the adsorption and desorption isotherms, respectively. The samples were activated at 70 °C under reduced pressure for 16 h prior to carry out the sorption measurements.

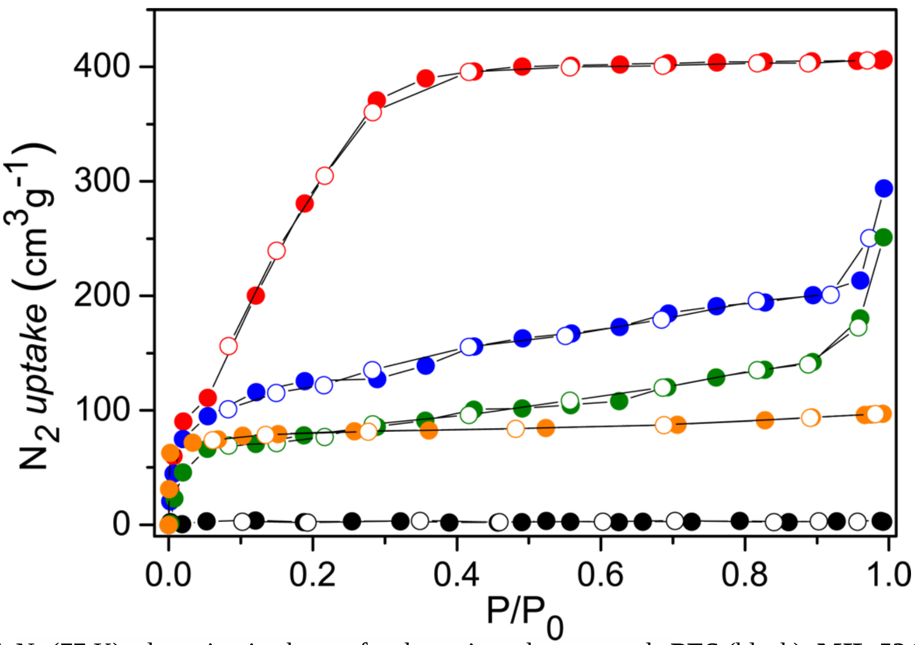

Figure S7. N_2 (77 K) adsorption isotherms for the activated compounds **PES** (black): **MIL-53@PES(Al)** (red); **MIL-53(Fe)@PES** (blue); **MIL-101(Fe)@PES** (green) and **SrCu$_6$Ser@PES** (orange). Filled and empty symbols indicate the adsorption and desorption isotherms, respectively. The samples were activated at 70 °C under reduced pressure for 16 h prior to carry out the sorption measurements.

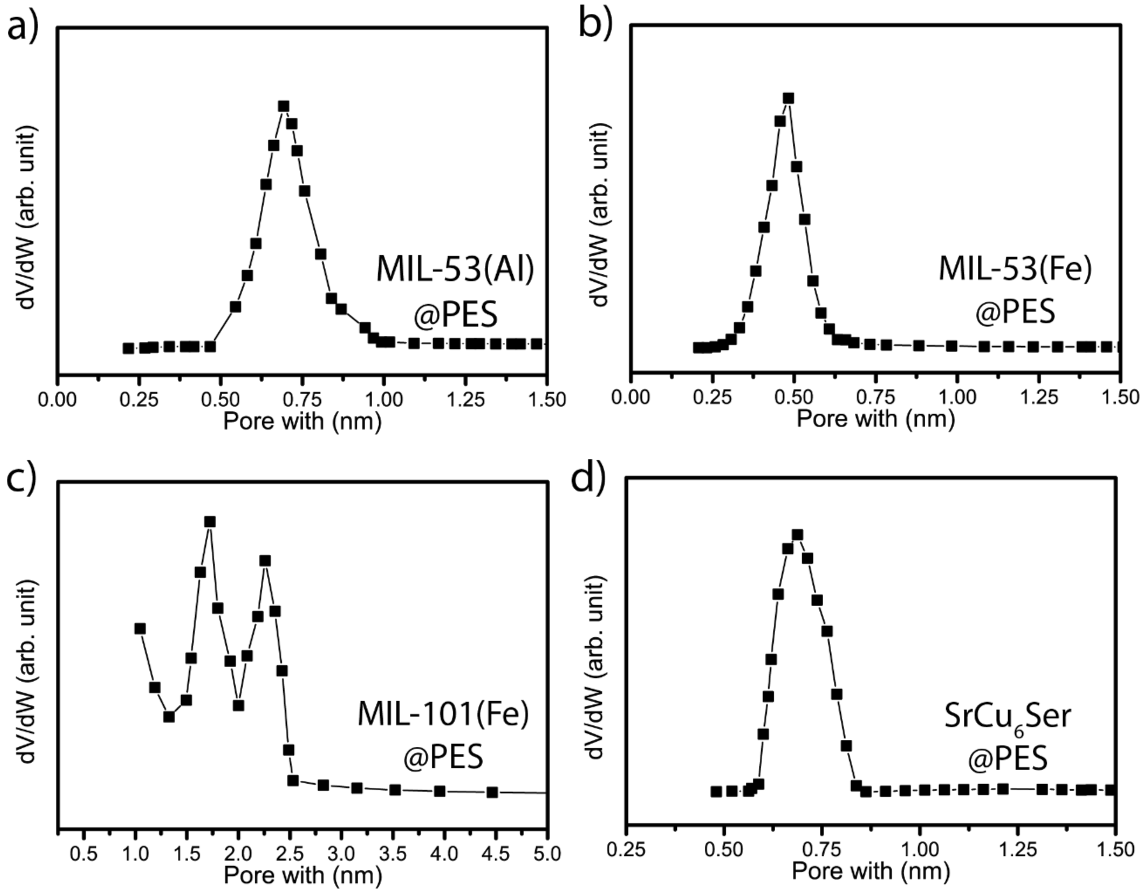

Figure S8. Pore size distribution of **MIL-53(Al)@PES** (a), **MIL-53(Fe)@PES** (b), **MIL-101(Fe)@PES** (c), and **$SrCu_6Ser$@PES** (d).

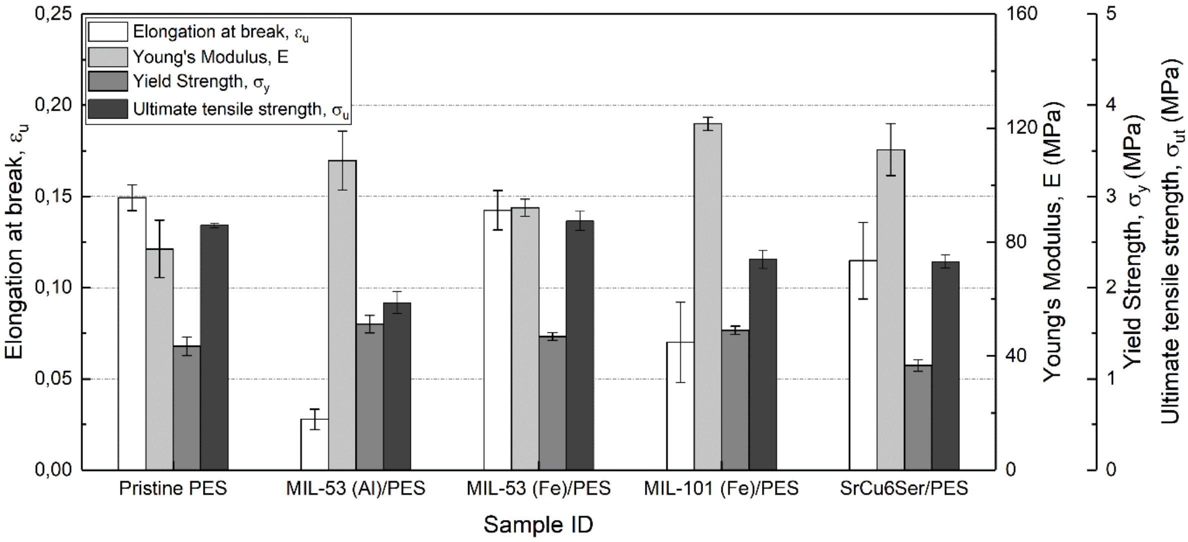

Figure S9. Comparison of significant macro-mechanical parameters between the investigated materials: Young's modulus (E) yield strength (σ_y) elongation to failure (ε_f) and ultimate tensile strength (σ_{ut}).

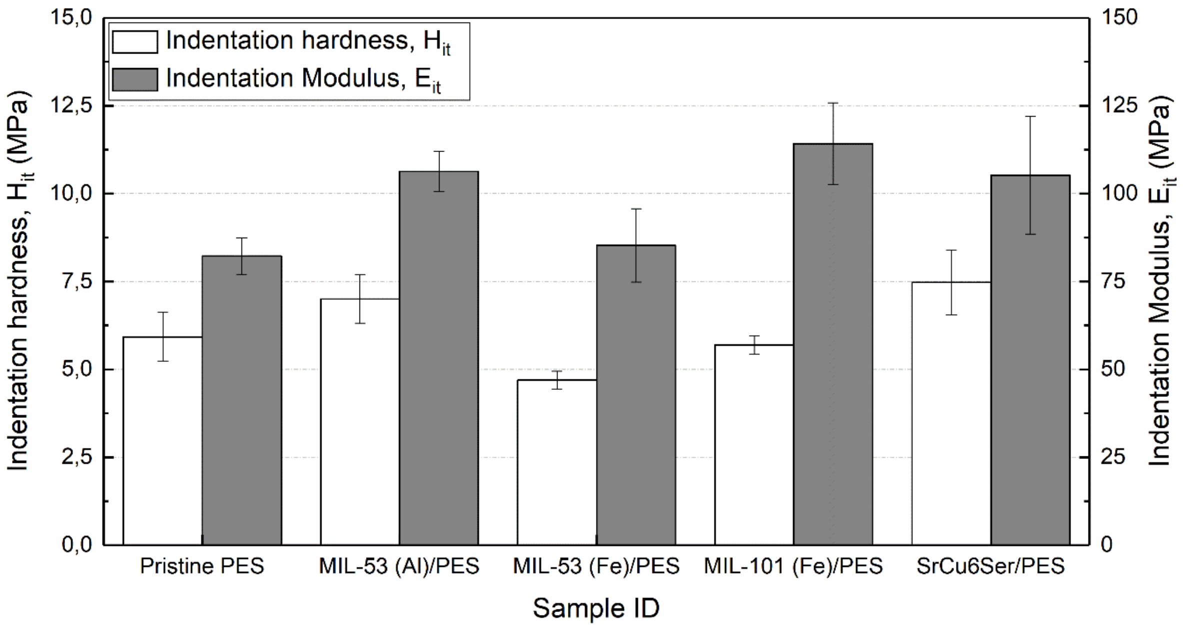

Figure S10. Comparison of significant nano-mechanical parameters between the investigated materials: indentation Young's modulus (E_{IT}) and nano-hardness (H_{IT}).

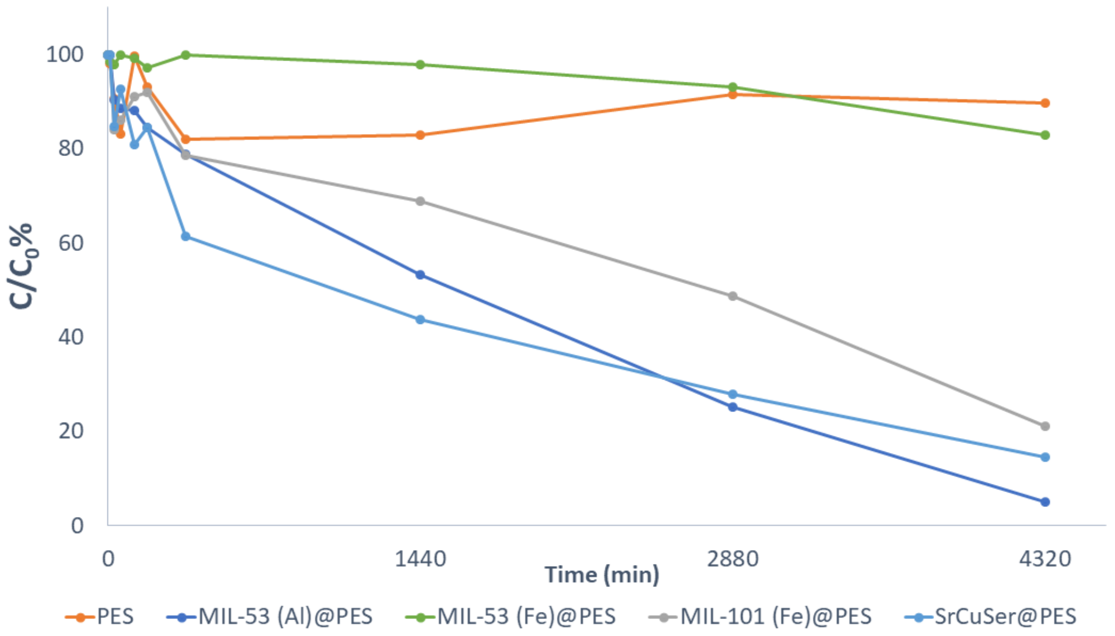

Figure S11. Variation of the concentration of Ni(II) ion *vs* time. Solid lines are a guide for eyes.

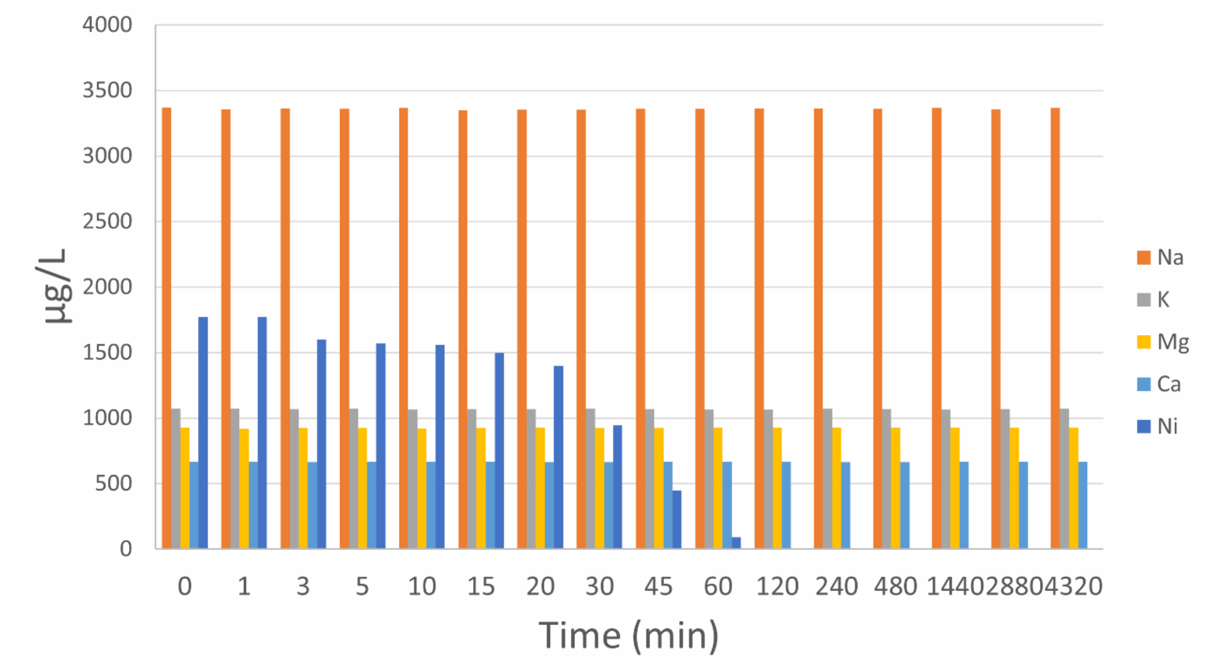

Figure S12. Variation of the concentration of common metal ions found in oligo-mineral water and Ni(II) cations added, with **MIL-53(Al)@PES** MMM *vs* time. Graphics are organized from data reported in Table S3.

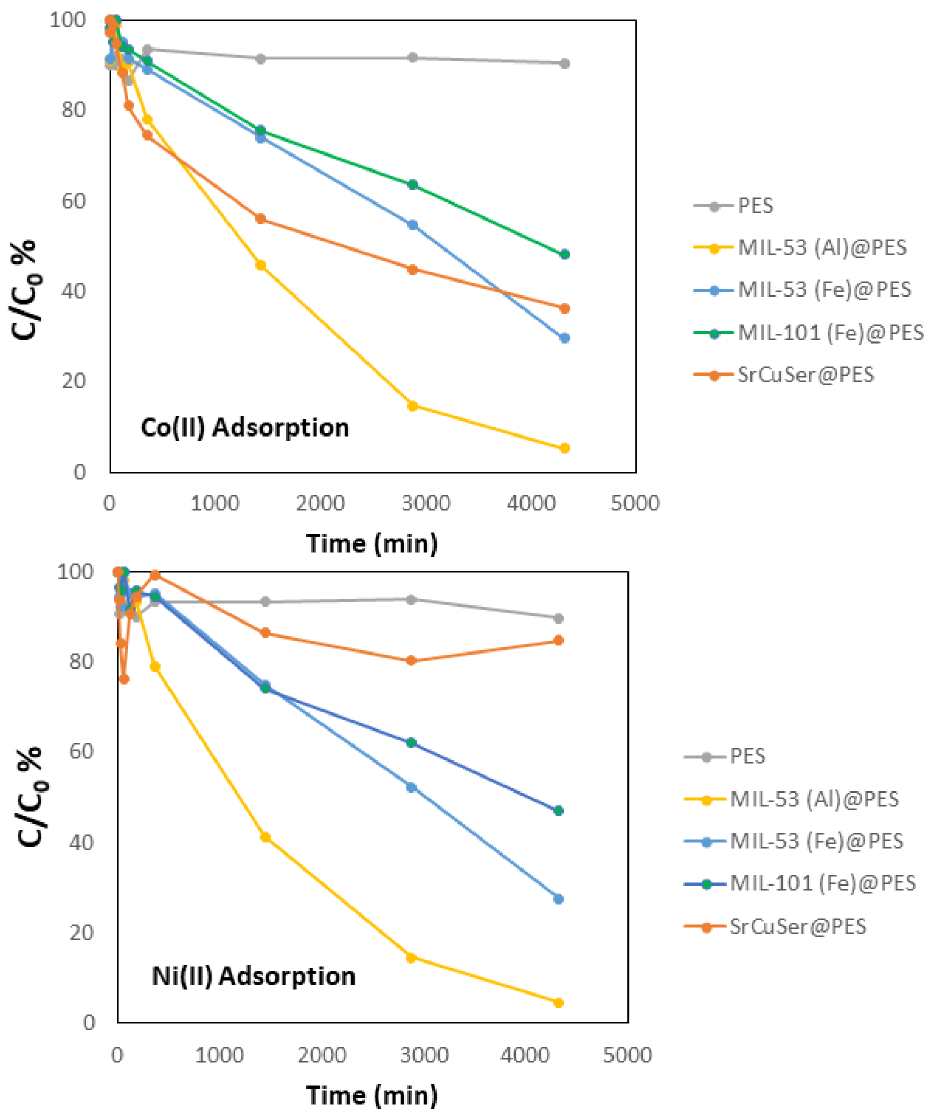

Figure S13. Variation of the concentration of Ni(II) and Co(II) ion *vs* time. Solid lines are a guide for eyes.

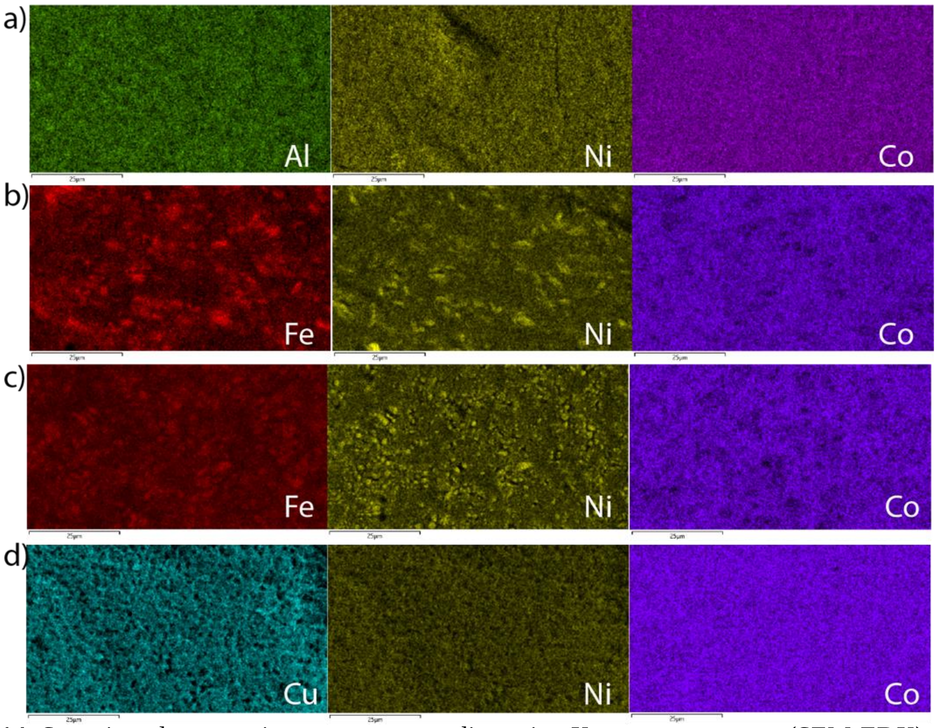

Figure S14. Scanning electron microscopy-energy dispersive X-ray spectroscopy (SEM-EDX) elemental mapping for a) **MIL-53(Al)@PES**; b) **MIL-53(Fe)@PES**; c) **MIL-101(Fe)@PES** and d) **SrCu$_6$Ser@PES**. First columns correspond to the main metal of the MOFs, second belongs to nickel and the third one to cobalt. Color code: aluminum, green; iron, red; copper, turquoise; nickel, gold and cobalt, purple.

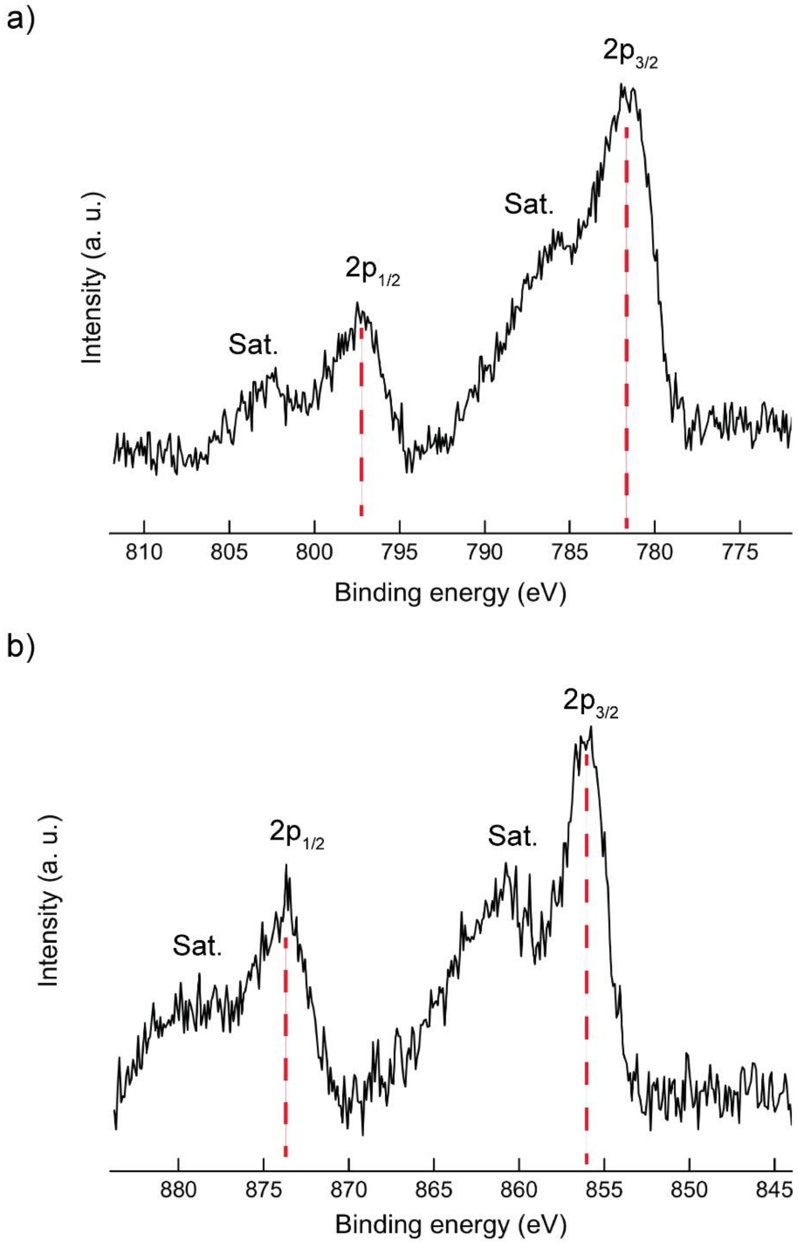

Figure S15. Co 2p (a) and Ni 2p (b) XPS spectra of **$SrCu_6$Ser@PES** after the capture experiments.

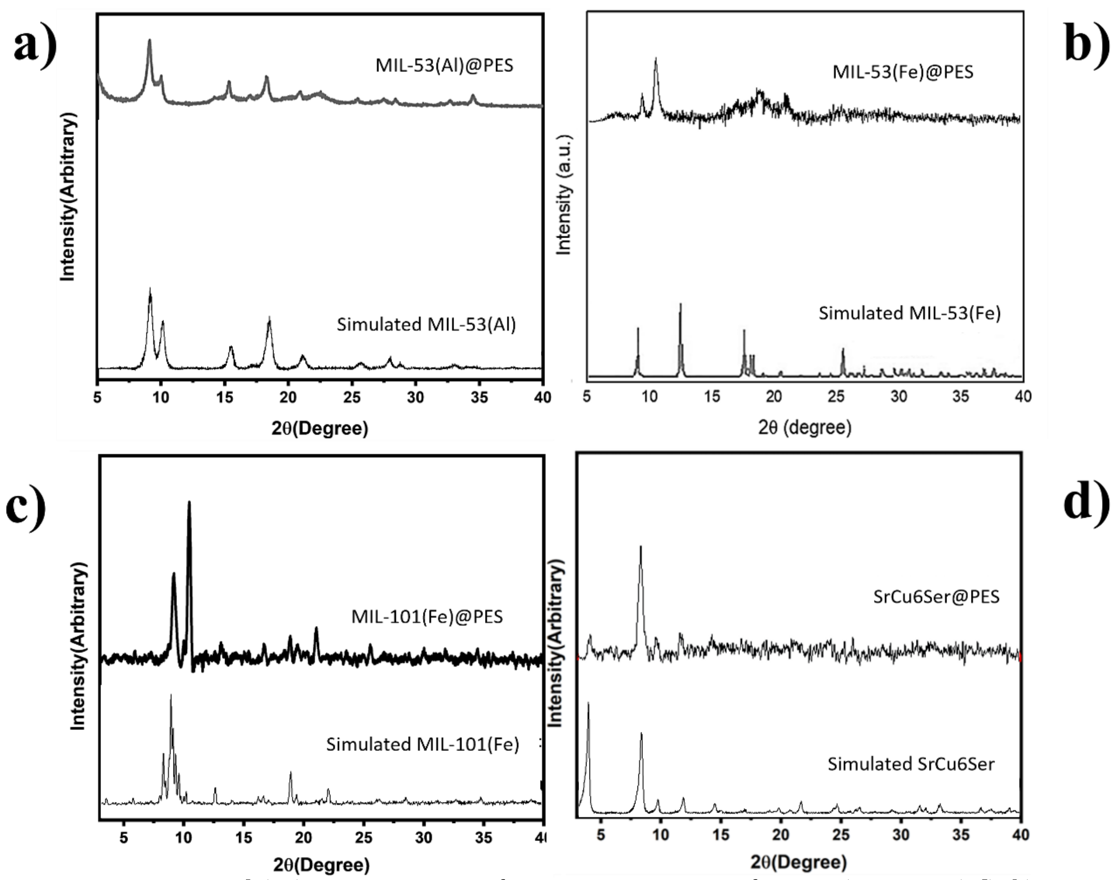

Figure S16. Experimental (up) PXRD patterns of MOF@PES MMMs after use a) **MIL-53(Al)**; b) **MIL-53(Fe)**; c) **MIL-101(Fe)** and d) **SrCu$_6$Ser**, compared with the calculated (down) PXRD patterns of the correspondent MOFs.

REFERENCES

(1) Rouquerolt, J.; Avnir, D.; Fairbridge, C. W.; Everett, D. H.; Haynes, J. H.; Pernicone, N.; Ramsay, J. D. F.; Sing, K. S. W.; Unger, K. K. Recommendations for the Characterization of Porous Solids. *Pure Appl. Chem.* **1994**, *66* (8), 1739–1758. https://doi.org/doi:10.1351/pac199466081739.
